# Identification of Virulence-Associated Properties by Comparative Genome Analysis of Streptococcus pneumoniae, S. pseudopneumoniae, S. mitis, Three S. oralis Subspecies, and *S. infantis*

**DOI:** 10.1128/mBio.01985-19

**Published:** 2019-09-03

**Authors:** Mogens Kilian, Hervé Tettelin

**Affiliations:** a Department of Biomedicine, Aarhus University, Aarhus, Denmark; b Department of Microbiology and Immunology, Institute for Genome Sciences, University of Maryland School of Medicine, Baltimore, Maryland, USA; Carnegie Mellon University; University of Leicester; University of Alabama at Birmingham

**Keywords:** *Streptococcus mitis*, *Streptococcus oralis*, *Streptococcus pneumoniae*, bacteremia, commensal, evolution, infective endocarditis, mutualism, virulence

## Abstract

From a common ancestor, Streptococcus pneumoniae and Streptococcus mitis evolved in parallel into one of the most important pathogens and a mutualistic colonizer of humans, respectively. This evolutionary scenario provides a unique basis for studies of both infection-associated properties and properties important for harmonious coexistence with the host. We performed detailed comparisons of 60 genomes of S. pneumoniae, S. mitis, Streptococcus pseudopneumoniae, the three Streptococcus oralis subspecies *oralis*, *tigurinus*, and *dentisani*, and Streptococcus infantis. Nonfunctional remnants of ancestral genes in both S. pneumoniae and in S. mitis support the evolutionary model and the concept that evolutionary changes on both sides were required to reach their present relationship to the host. Confirmed by screening of >7,500 genomes, we identified 224 genes associated with virulence. The striking difference to commensal streptococci was the diversity of regulatory mechanisms, including regulation of capsule production, a significantly larger arsenal of enzymes involved in carbohydrate hydrolysis, and proteins known to interfere with innate immune factors. The exclusive presence of the virulence factors in S. pneumoniae enhances their potential as vaccine components, as a direct impact on beneficial members of the commensal microbiota can be excluded. In addition to loss of these virulence-associated genes, adaptation of S. mitis to a mutualistic relationship with the host apparently required preservation or acquisition of 25 genes lost or absent from S. pneumoniae. Successful adaptation of S. mitis and other commensal streptococci to a harmonious relationship with the host relied on genetic stability and properties facilitating life in biofilms.

## Abstract (precis)

Streptococcus pneumoniae is one of the most important human pathogens but is closely related to Streptococcus mitis, with which humans live in harmony. The fact that the two species evolved from a common ancestor provides a unique basis for studies of both infection-associated properties and properties important for harmonious coexistence with the host. By detailed comparisons of genomes of the two species and other related streptococci, we identified 224 genes associated with virulence and 25 genes unique to the mutualistic species. The exclusive presence of the virulence factors in S. pneumoniae enhances their potential as vaccine components, as a direct impact on beneficial members of the commensal microbiota can be excluded. Successful adaptation of S. mitis and other commensal streptococci to a harmonious relationship with the host relied on genetic stability and properties facilitating life in biofilms.

## INTRODUCTION

The Mitis group of the genus *Streptococcus* includes several phylogenetic clusters, one of which is composed of the three species Streptococcus pneumoniae, Streptococcus pseudopneumoniae, and Streptococcus mitis, all with a primary habitat in the human upper respiratory tract (1–3). In spite of their close genetic relationship, the three species demonstrate strikingly different pathogenic potentials. S. pneumoniae is one of the most important human pathogens. It causes local infections in the lungs and middle ear and is capable of active penetration to the bloodstream to cause bacteremia and disseminated infection, including purulent meningitis (3, 4). S. mitis, in contrast, is an important member of the commensal microbiota of the oral cavity and pharynx. If it gains access to the bloodstream via a compromised barrier, e.g., during a period of inflamed periodontium or in connection with dental procedures, S. mitis may cause bacteremia and/or infective endocarditis in predisposed individuals (5–8). The more recently described *S. pseudopneumoniae* appears to be an intermediary, both genetically and functionally, of the two other species.

Other species genetically related to but distinct from the S. pneumoniae-S. mitis*-S. pseudopneumoni*ae cluster are Streptococcus oralis and Streptococcus infantis, which both constitute a proportionally important part of the oral commensal microbiota. A recent study reported that S. oralis, but not S. mitis, is frequently isolated from great apes, although isolates clustered on separate lineages outside the main cluster of human S. oralis strains, suggesting that S. oralis is part of the commensal flora in higher primates and evolved prior to humans (2). S. oralis includes three subspecies, i.e., subspecies *oralis*, *tigurinus*, and *dentisani*, of which the latter previously was classified as S. mitis biovar 2 (1).

Numerous studies, both focused and large scale, have been performed to identify virulence factors of S. pneumoniae. These studies include *in vitro* studies of single properties, experimental infection studies in mice challenged with targeted knockout mutants or mutants generated by large-scale signature-tagged mutagenesis (STM), and analyses of gene expression *in vivo* during experimental infections (3, 4, 9–15). However, three STM studies (10–12) using experimental mouse models of pneumonia and bacteremia showed surprisingly limited overlap of results (14). Likewise, genes identified as attenuated for virulence in such studies are not necessarily upregulated or may even be downregulated in transcription studies performed during experimental infections (14). However, there is consensus about the importance of a number of properties. The key virulence function of the capsular polysaccharide is supported by numerous studies, including evidence gained from population-based vaccination (3, 4). Other virulence properties include the IgA1 protease, hyaluronidase, pneumolysin, the major autolysin, and pneumococcal surface protein A, which appear to be indispensable in experimental pneumonia and bacteremia in mice. However, our recent comprehensive screening demonstrated that most members of the commensal species also express capsular polysaccharides, some structurally identical to recognized serotypes of pneumococci (16). It is yet unclear if these capsules reach the same size as those expressed by pneumococci, which potentially would affect their antiphagocytic properties. Likewise, genes encoding some of the aforementioned proteins may also be found in members of the commensal species (17–19). Thus, although S. pneumoniae probably is one of the most studied bacterial pathogens, the mechanism underlying its capability to transition from colonizer to pathogen remains unclear.

We previously presented evidence to support the evolutionary scenario that the populations of S. mitis and S. pneumoniae evolved from a common ancestor similar to today’s pneumococcus. In an attempt to survive the evolutionary bottleneck caused by a shortage of potential hosts, the vast majority of lineages, now recognized as S. mitis, eliminated genes that challenged their host, thereby securing immune tolerance, sustained colonization, and mainly vertical spreading. The few that became today’s S. pneumoniae potentiated their ability to survive by horizontal spreading among individuals, a strategy that subsequently proved successful in parallel with the increasing size and density of the human population, as reflected in the recent burst of the pneumococcal population (20). This evolutionary model is consistent with the variable numbers of virulence factor genes in S. mitis strains and their long-term sequence diversification, which excludes that they are a result of recent transfer from pneumococci (20). During the parallel evolution of the two populations, the pneumococcus optimized its ability to adapt to new hosts and environmental selection pressures, including host immune responses and antibiotics, by frequent import of genes from other pneumococci and members of the genetically related commensal species. The reductive evolution of the S. mitis genome, in contrast, eliminated genes that may challenge the host and, in addition, secured genetic stability (20).

This evolutionary scenario provides a unique basis for studies of both infection-associated properties and properties important for harmonious colonization. In this study, we performed detailed comparisons of 60 genome sequences of strains assigned to the species S. pneumoniae, S. mitis, *S. pseudopneumoniae*, S. oralis subspecies *oralis*, *tigurinus*, and *dentisani*, and *S. infantis*. This strategy enabled us to identify genes exclusively present in the virulent S. pneumoniae or the related mutualistic species. Based on information concerning the clinical origin of S. mitis strains, we were furthermore able to address the question of potential differences in pathogenic potential related to variably present properties associated with virulence in pneumococci.

Although the pneumococcus colonizes a significant part of the human population, especially children in daycare settings, without causing clinical disease, colonization by the individual clones challenges the host and induces an immune response that eventually results in elimination after a limited colonization period (21). Unlike most commensals, potential pathogens like S. pneumoniae have the capacity to strongly interact with host cells and tissues, thereby inducing proinflammatory components of the innate immune system (22). In contrast, clones of the species S. mitis, S. oralis, and *S. infantis* may colonize the host during its entire life span, although fluctuations in relative proportions may occur, probably due to internal competition between individual clones (23). As long as they remain in their natural habitat, the immune system reacts with tolerance. Throughout the manuscript, we therefore use the term commensal or mutualist for viridans streptococci other than pneumococci.

## RESULTS

### Presentation of genomes.

**FIG 1:**
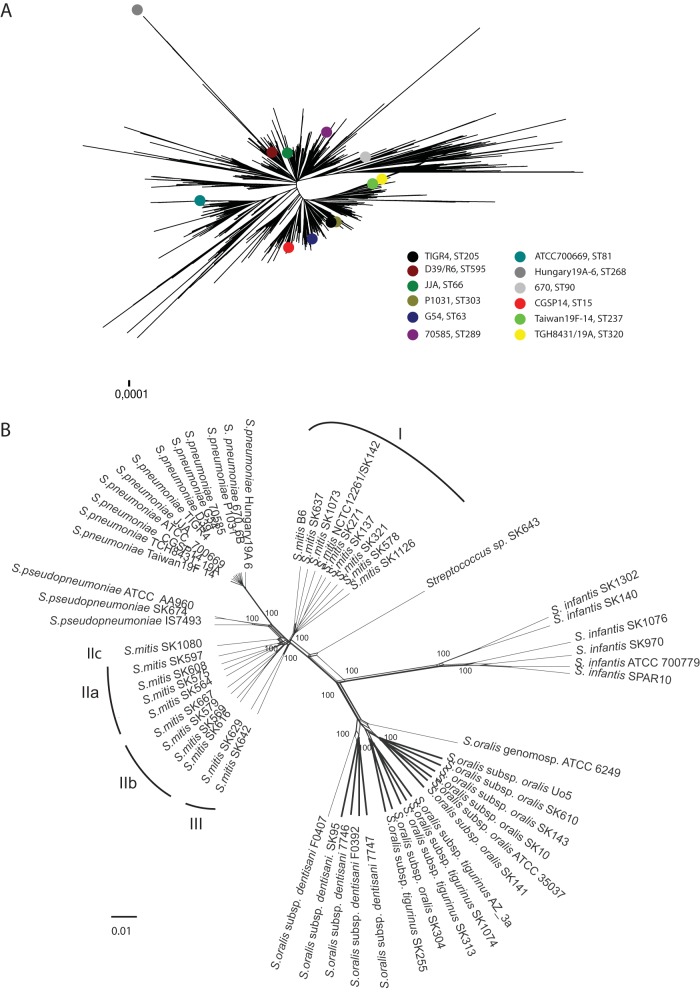
(A) Minimum evolution tree based on concatenated sequences of MLST loci showing the representability of the 13 S. pneumoniae indicated by colored dots. (B) Evolutionary relationships of genomes inferred from SplitsTree analysis. Phylogenetic trees of 60 genomes based on core sequence of 844,932 bp shared by all strains in the collection. The tree shows three major clusters supported by bootstrap values of 100, one consisting of S. mitis, S. pneumoniae, and *S. pseudopneumoniae*, a second composed of S. oralis (subsp. *oralis*, subsp. *dentisani*, subsp. *tigurinus*, and a genomosubspecies), and a third consisting of strains of *S. infantis*. The 20 S. mitis strains are segregated into two distinct clusters, one of them consisting of several minor subclusters.

Table S1A in the supplemental material shows the size and completion status of the 60 genomes included in the core of the study together with accession numbers and references. The S. pneumoniae strains represented all gap-free genomes available. According to the phylogenetic tree based on concatenated sequences of seven MLST genes (https://pubmlst.org/spneumoniae/), they provide a fair representation of the population of pneumococci (Fig. 1A). Other species were represented by the respective designated type strains and representative strains mainly from the SK collection of M. Kilian. The average sizes (in megabases) of the genomes of the individual species were the following: S. pneumoniae, 2.14 ± 0.07; *S. pseudopneumoniae*, 2.12 ± 0.06; S. mitis, 2.01 ± 0.095; S. oralis, 1.95 ± 0.09; and *S. infantis*, 1.83 ± 0.088. The average number of coding DNA sequences (CDS) was the following: S. pneumoniae, 2,140 ± 107; *S. pseudopneumoniae*, 2,105 ± 58; S. mitis, 1,913 ± 96; S. oralis, 1,902 ± 103; and *S. infantis* 1,868 ± 232.

10.1128/mBio.01985-19.6 Table S1 (A) Genomes of *Streptococcus* strains included in the study. (B) DNA restriction modification systems in the examined *Streptococcus* genomes. (C) Two-component system (TCS) response regulators in S. pneumoniae and related species. (D) Transcriptional regulator genes in S. pneumoniae TIGR4 and their presence in other strains of the study. (E) Carbohydrate uptake systems in S. pneumoniae and their presence in related species. (F) ABC transporters other than carbohydrate transporters specific to S. pneumoniae. Download Table S1, PDF file, 0.3 MB. Copyright © 2019 Kilian and Tettelin. 2019 Kilian and Tettelin This content is distributed under the terms of the Creative Commons Attribution 4.0 International license.

### Genomes finished as part of this study.

Figure S1 provides a graphic summary of the completed genomes of the type strain of S. mitis NCTC12261 (SK142) and S. mitis strain SK637. The sizes of the two gap-free genomes were 1,868,883 and 1,942,107 bp, respectively, which is within the range of other S. mitis genomes (Table S1). The number of CDS in the two genomes was 1,741 and 1,816, respectively. Compared to the only other S. mitis genome that was completed, strain B6 (24), the two new genomes are significantly smaller (13% and 9.5%) and include fewer CDS (13% and 9.3%). The circular diagram presented in Fig. S1A illustrates that both genomes share a majority of CDS with other streptococci, including the species S. oralis, S. pneumoniae, and *S. pseudopneumoniae* (third inner rim, labeled Core). The two S. mitis genomes display a symmetrical GC-skew profile (G−C/G+C) typical of low-GC Gram-positive species, where ∼50% of the GC-skew function is positive starting at the *dnaA* gene and the other half is negative (Fig. S1B). The orientation of CDS predominantly follows the GC-skew, another hallmark of low-GC Gram-positive species.

10.1128/mBio.01985-19.1 FIG S1 Summary of genome properties of the completed genomes of S. mitis NCTC12261 (type strain) and SK637. (A) The outer rim displays a GC% bar graph in red (the dashed line shows the maximum value). The next two rims show CDS predicted on the forward and reverse strands, green bars indicate rRNAs, and red bars indicate tRNAs. The next two rims (rims 4 and 5 from the outside) indicate core genes that are shared by all seven strains included in the display (including the reference) and unique genes that are present only in the reference genome (NCTC 12261 or SK637). The sixth innermost rims indicate genes that are shared by the strain noted on the rim and the reference strain. (B) The two S. mitis genomes display a symmetrical GC-skew profile (G-C/G+C) typical of low-GC Gram-positive species, where ∼50% of the GC-skew function is positive starting at the *dnaA* gene and the other half is negative. Download FIG S1, PDF file, 0.4 MB. Copyright © 2019 Kilian and Tettelin. 2019 Kilian and Tettelin This content is distributed under the terms of the Creative Commons Attribution 4.0 International license.

### Phylogenetic analysis and sequence-based identification.

The whole-genome multiple alignment performed using the automated CloVR-Comparative pipeline (25) showed that sequences shared by all 60 genomes belonging to S. pneumoniae, S. mitis, S. oralis subsp. *oralis*, S. oralis subsp. *tigurinus*, S. oralis subsp. *dentisani*, *S. infantis*, and the two related singleton strains (ATCC 6249 and SK643) amounted to 844,932 nucleotides (nt) per genome. The average %G+C of the core genomes of the individual species was the following: S. pneumoniae, 42.3 ± 0.1; *S. pseudopneumoniae*, 42.5 ± 0.06; S. mitis, 42.4 ± 0.28; S. oralis, 43.1 ± 0.3; and *S. infantis*, 41.2 ± 0.2. The number of polymorphic sites within the 844,932-bp core sequence amounted to 312,931 (37%), of which 258,625 sites were parsimoniously informative. These concatemers of sequences shared by all strains were used to generate minimum evolution phylogenetic trees using MEGA 7.0.26 (26) and SplitsTree4 (version 4.14.4) (27).

The tree generated in SplitsTree (Fig. 1B) shows three major clusters supported by bootstrap values of 100, one consisting of S. mitis, S. pneumoniae, and *S. pseudopneumoniae*, a second composed of S. oralis (subsp. *oralis*, subsp. *dentisani*, subsp. *tigurinus*, and a singleton representing a genomosubspecies), and a third consisting of strains of *S. infantis.* The analysis shows clear evidence of recombination between the lineages. The tree reveals segregation of the 20 S. mitis strains into three major clusters. One cluster consisted of nine strains clearly separated from the remaining strains by a common stalk. The second cluster consisted of the remaining eleven strains, which formed three less well-defined subclusters, and the third consisted of only two strains. The three main S. mitis clusters will be referred to as clusters I to III and the tentative subclusters in cluster II as IIa to IIc, as shown in Fig. 1. As noted before (1), strain SK643 was distant from all other strains in the collection and is referred to as an unnamed species pending the detection of additional strains. The same topology was detected in phylogenetic trees generated in MEGA7 using the minimum evolution or maximum parsimony algorithm (not shown).

### Comparison of genome structure.

**FIG 2:**
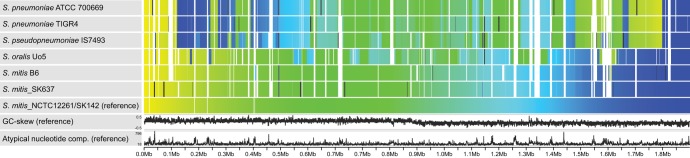
Synteny gradient display visualizing changes in synteny of seven representative genomes relative to the reference, S. mitis NCTC12261. The reference genome’s genes are colored from yellow to blue on a gradient from left to right. The figure reveals conservation of the color gradient in syntenic regions, while shared genes located in rearrangements show up as color mismatches between the reference and query genomes. Gaps in query genomes (white spaces) represent genes that are present in the reference but not the query. Query genes with paralogs are displayed in black. The figure reveals conservation of gene synteny between the three gap-free S. mitis genomes. Synteny is still mostly conserved with S. oralis Uo5, although blocks of genes breaking the color gradient appear. Given that the leftmost blocks are dark blue and the rightmost ones are light green, it is likely that these nonsyntenic blocks are the results of inversions that are symmetrical with the chromosome’s start (or terminus) of replication. Two successive nested inversions will leave the tips of the first inversion in place (breaks of color gradient), while the second inversion will restore the inner part of the gradient. Many such nested symmetric inversions then leave many nonsyntenic blocks of different colors. Similar inversions are observed between the S. mitis reference and S. pneumoniae and *S. pseudopneumoniae* genomes, but syntenic blocks remain larger, indicative of fewer inversion events and the close evolutionary relationship.

The Sybil-generated synteny gradient display (28), which enables the visualization of changes in synteny relative to a reference, is shown in Fig. 2. In this view, a reference genome’s genes are colored from yellow to blue on a gradient from left to right. If a query genome shares a gene in a cluster of syntenic orthologs (29) with a reference gene, then it is drawn above the matching reference gene but using a color that corresponds to the query gene’s position in its native genome. The resulting figure reveals conservation of the color gradient in syntenic regions, while shared genes located in rearrangements will appear as breaks in the color gradient between the reference and query genomes. Gaps in query genomes (white spaces) represent genes that are present in the reference but not the query. Query genes with paralogs are displayed in black. Figure 2 reveals conservation of gene synteny between the three gap-free S. mitis genomes selected for this display and evidence of a shared inversion relative to S. pneumoniae and *S. pseudopneumoniae* genomes. As the inversion is symmetrical with respect to the origin of replication, the GC-skew is not affected.

### Core genes.

The number of core genes (clusters of syntenic orthologs [29] present in all 60 genomes) was 690. The number was 894 when only S. mitis, S. pneumoniae, *S. pseudopneumoniae*, and the three subspecies of S. oralis were included. When only S. mitis, S. pneumoniae, and *S. pseudopneumoniae* were included, the core genome consisted of 995 genes, again indicative of their evolutionary relationship.

### Regions of diversity.

Previous comparative genomic analyses of S. pneumoniae identified 13 loci, termed regions of diversity (RD1 to RD13), that consist of 4 to 37 genes (30, 31). Of the 13 RDs, seven have been associated with atypical GC content and eight have been determined to be flanked by insertion sequences or remnants of mobile genetic elements, suggesting that they were acquired by horizontal transfer (Table S2). The individual RDs were present in one to nine of the S. pneumoniae strains examined in this study. The genes of RD2, RD6, and RD7 were specific to TIGR4. Among the remaining RDs, four (RD4, RD5, RD9, and RD12) were present in five, four, seven, and five of the 13 pneumococcal genomes, respectively, but were completely absent from the commensal strains. Genes contained in RD1, RD3, RD8, RD10, RD11, and RD13 were present in two, 12 (different versions of *cps* locus according to serotype), seven, six, eight and nine pneumococcal genomes and showed variable presence in the collection of commensal strains (Table S2).

10.1128/mBio.01985-19.7 TABLE S2 Characteristics of regions of diversity and their occurrence in the genomes of strains of S. pneumoniae and commensal species. Download Table S2, XLSX file, 0.01 MB. Copyright © 2019 Kilian and Tettelin. 2019 Kilian and Tettelin This content is distributed under the terms of the Creative Commons Attribution 4.0 International license.

RD1 contains genes encoding a choline-binding protein (CbpI), zinc metalloproteinase C (ZmpC), an acetyltransferase, and five hypothetical proteins. The locus was also present in five strains of S. mitis (NCTC12261, SK608, SK637, SK667, and SK1126) but not in any of the other species.

RD3 constitutes the capsular biosynthesis locus (*cps*). The locus is present in all pneumococcal strains, apart from the unencapsulated R6 strain, with four conserved regulatory genes but otherwise distinct combinations of genes according to the respective capsular serotypes. In accordance with our recent findings (16), a complete capsular biosynthesis operon also was found in all examined commensal strains except for the three strains of *S. pseudopneumoniae* and four of the 20 strains of S. mitis. While the locus in the S. pneumoniae strains was flanked by transposases (often degenerated), this was not the case in the commensal strains.

The 34,119-bp region RD8 in TIGR4 encodes 37 genes, including complete V-type ATPase, an oxidoreductase, a putative neuraminidase, *N*-acetylneuraminate lyase, a putative *N*-acetylmannosamine-6-P epimerase, a putative phosphosugar-binding transcriptional regulator, type II DNA modification methyltransferase Spn5252IP, a toxin-secreting ABC transporter, and several hypothetical proteins. The complete range of genes is unique to TIGR4 in our collection, while six additional S. pneumoniae strains carried the RD8a part (predicted operon nomenclature according to Obert et al. [32]) of the region. S. pneumoniae strain 70585 carried the RD8b2 operon but not RD8a. S. mitis strain B6 carries genes corresponding to RD8a (SP_1315 to SP_1331 [termed SP_1315-1331] minus SP_1326). Six additional S. mitis strains and six S. oralis strains carried genes corresponding to SP_1333, SP_1334, and SP_1336.

RD10 contains the gene encoding the largest surface protein (4,776 aa) detected in S. pneumoniae (PsrP) and eight glycosyltransferase genes responsible for its glycosylation. The presence of this region is described below in the context of LPxTG-anchored proteins.

The four genes that constitute RD11 encode UDP-glucose 4-epimerase, galactose-1-phosphate uridylyltransferase, a PhoU family transcriptional regulator, and a hypothetical protein. They are present in eight of the 13 S. pneumoniae strains, in the three *S. pseudopneumoniae* strains, and in four S. mitis strains.

RD13 in TIGR4 is composed of nine genes involved in fucose metabolism and transport by a four-component phosphotransferase system (PTS). This pattern is shared by nine of the 13 S. pneumoniae strains and by S. mitis strains SK597 and SK608. In four of the S. pneumoniae strains (CGSP14, 670, G54, and 70585), nine genes include the same initial fucose isomerase combined with distinct genes encoding fuculose phosphate aldolase, endo-β-galactosidase, α-galactosidase, and a three-component sugar ABC transporter. This pattern was not present in any of the commensal strains.

### DNA restriction-modification systems.

The S. pneumoniae TIGR4 and D39 genomes were reported to contain two type I, three type II, and one type IV restriction-modification (RM) systems (33, 34). The six-gene type I RM system (*ivr*) in strain D39, termed SpnD39III (SPD_0450-0455 corresponding to SP_0504-0510), was recently shown to undergo switching into alternative specificities with distinct methylation patterns that affect expression of genes, including those responsible for capsular polysaccharide production (35). Our detailed analyses showed that only S. pneumoniae strains contained the complete SpnD39III locus, although four of the 13 S. pneumoniae strains (Taiwan19F, 70585, G54, and CGSP14) apparently lacked an ortholog of the phage integrase family protein SPD_0452/SP_0506. The three *S. pseudopneumoniae* strains had three of the genes, i.e., *hdsS*, *hdsM*, and *hdsR* (SPD_0452-0455), which would exclude a role in phase variation.

The additional type 1 RM system (*tvr*), spanning the genes SP_0886 to SP_0892 in TIGR4, was detected in all strains of S. pneumoniae and *S. pseudopneumoniae*, in some strains of the three S. oralis subspecies, and in *S. infantis* SK1302 (Table S1B) but not in any of the S. mitis strains. In all the positive strains, except for S. pneumoniae strains D39 and R6, the genes of this type 1 RM system locus (subunits R, M, and S) were interspersed with phage integrase genes, genes encoding an AbrB family transcriptional regulator, a death-on-curing protein, and, in some strains, an additional restriction endonuclease or modification gene (Fig. S2A).

10.1128/mBio.01985-19.2 FIG S2 Type 1 RM systems in streptococcal genomes. (A) The type 1 RM system (*tvr*) spanning SP_0886-SP_0892 in TIGR4 was present in all strains of S. pneumoniae and *S. pseudopneumoniae*, in some strains of the three S. oralis subspecies, and in *S. infantis* SK1302 (Table S2) but not in any of the S. mitis strains. The locus was interspersed with phage integrase genes, genes encoding an AbrB family transcriptional regulator, a death-on-curing protein, and, in some strains, an additional restriction endonuclease or modification gene. (B) The unrelated type I RM system in S. mitis NCTC12261 (SM12261_1408-1413) was present in the majority of commensal species, except for strains of *S. pseudopneumoniae* and three strains of S. mitis. The locus showed a remarkable strain-specific organization and degree of completeness. Download FIG S2, PDF file, 0.6 MB. Copyright © 2019 Kilian and Tettelin. 2019 Kilian and Tettelin This content is distributed under the terms of the Creative Commons Attribution 4.0 International license.

10.1128/mBio.01985-19.8 TABLE S3 Teichoic acid biosynthesis genes. Download Table S3, XLSX file, 0.2 MB. Copyright © 2019 Kilian and Tettelin. 2019 Kilian and Tettelin This content is distributed under the terms of the Creative Commons Attribution 4.0 International license.

With a few exceptions, the three strains of *S. pseudopneumoniae* had the same sets of other RM systems as S. pneumoniae, whereas the three commensal species lacked orthologs of most of them (Table S1B). Apart from a few exceptional strains, S. mitis strains lacked the entire range of RM systems present in S. pneumoniae. Only the type IV endonuclease was present in many of the members of the commensal species (Table S1B). In the genomes of the three *S. pseudopneumoniae* strains, upstream of the truncated SPD_1630 homolog, an additional putative restriction modification set of genes encoding a methyltransferase (HMPREF1112_RS07700) and a restriction endonuclease (HMPREF1112_RS07695) were identified. These were present also in S. pneumoniae ATCC 700669.

A comprehensive search for putative RM system genes in S. mitis revealed a complete type I system (subunits R, M, and S) in S. mitis NCTC12261 (SM12261_1408-1413) unrelated to the pneumococcal type I systems. The locus was present in the majority of commensal species except for strains of *S. pseudopneumoniae* and three strains of S. mitis. The locus showed a remarkable strain-specific organization of the subunit genes and degree of completeness, as well as in the number of accessory genes, many of which encoded hypothetical proteins. In one strain of S. mitis (SK575), the type I RM system was replaced by a homolog of an EcoDLXXI type IV restriction-modification methylase (Fig. S2B), with 87% nucleotide identity to a gene in Streptococcus thermophilus. In addition, all S. mitis strains save five (NCTC12251, B6, SK137, SK564, and SK597) possessed from one to four type III RM systems not present in S. pneumoniae. Finally, four S. mitis strains (B6, SK321, SK667, and SK1126) had one type II system.

### Two-component signal transduction systems.

Genomes of S. pneumoniae strains contain 13 two-component histidine transcriptional regulator genes in addition to the DNA-binding response regulator, SP_0376, which is not associated with a histidine kinase (33, 36). The only exception among the 13 S. pneumoniae strains examined is CGSP14, which lacks SP_0155/SP_0156 orthologs. With two notable exceptions, these sets of response regulator genes are also present in the commensal species (Table S1C). The exceptions are that all strains of the commensal species lack homologs of the two phosphate (Pho) family regulators SP_2082/SP_2083 (PnpR/S) and SP_2192/SP_2193 (TCS06). According to Novak et al. (37), PnpR/S controls the expression of pneumococcal surface antigen PsaA (SP_0117), and TCS06 regulates expression of choline-binding protein A (CbpA; SP_2190), both of which are also lacking in the commensal species. An ortholog of TCS06, however, was present in all five strains of S. oralis subspecies *tigurinus*, whereas CbpA was not.

10.1128/mBio.01985-19.9 TABLE S4 Presence of the 224 genes identified as specific to S. pneumoniae and 25 genes specific to commensal streptococci in 7,617 streptococcal genome assemblies, as determined by BLASTN alignment (see Materials and Methods). Download Table S4, XLSX file, 0.03 MB. Copyright © 2019 Kilian and Tettelin. 2019 Kilian and Tettelin This content is distributed under the terms of the Creative Commons Attribution 4.0 International license.

### Transcriptional regulators.

The genome of S. pneumoniae TIGR4 contains 23 transcriptional regulators located in association with various operons, such as PTS (SP_0246/7, SP_0306, SP_0330, SP_0473, SP_1856, SP_1899, and SP_2020), ABC transporters (SP_0716, SP_1057, SP_1433, SP_1821, SP_1920, SP_2088/90, and SP_2172), teichoic acid biosynthesis (SP_1942), type II restriction-modification system (SP_1936), tryptophan synthesis (SP_1809), ion transport (SP_1227), oxidoreductase (SP_0789), thioredoxin (SP_1774), lipoprotein signal peptidase (SP_0927), or unknown functions (SP_0191, SP_1989, and SP_1946) (33). With the exception of SP_1057, they were present in all or in the majority of the 13 pneumococcal strains (Table S1D).

10.1128/mBio.01985-19.10 TABLE S5 Selected properties of strains assigned to subclusters of S. mitis according to the phylogenetic tree in Fig. S5B. Download Table S5, XLSX file, 0.02 MB. Copyright © 2019 Kilian and Tettelin. 2019 Kilian and Tettelin This content is distributed under the terms of the Creative Commons Attribution 4.0 International license.

Some of the transcriptional regulators were shared with *S. pseudopneumoniae*, whereas most were absent from strains of S. mitis and other commensal streptococci in correspondence with the presence or absence of the respective associated operons (Table S1D). Interestingly, the transcriptional regulator gene SP_1920 (multiple antibiotic resistance regulator; MarR family), associated with an ABC transporter, is a pseudogene in all strains of S. pneumoniae but is intact in all other strains.

### Virulence-associated genes.

To identify genes that may be important for the different pathogenic potentials of S. pneumoniae and the closely related commensal species, we searched for genes that were present in at least 11 out of the 13 genomes of S. pneumoniae and present in 20% or less of the 20 strains of S. mitis using the annotated genome of TIGR4 as a reference. Intact or degenerate transposase/insertion sequences, which are almost exclusively present in S. pneumoniae (20), were excluded from the examination. A total of 224 genes fulfilled these criteria (Table 1). Of these, 29 encoded proteins are still annotated as hypothetical. Among the 224 genes, 115 were absent from all S. mitis strains. Of these, 77 were also absent from S. oralis strains, and 49 genes were absent from all 40 strains of S. mitis, S. oralis, and *S. pseudopneumoniae*. Many of the genes that constitute the differences between S. pneumoniae and the commensal species are part of putative operons of 2 to 16 genes in S. pneumoniae. In total, the genes identified constituted 101 functional loci (some already mentioned above), in which all component genes were either absent from or present in the individual strains. The following sections focus on selected properties that are traditionally associated with virulence in S. pneumoniae and genes that, according to the present study, are present in S. pneumoniae and absent from S. mitis.

**TABLE 1:** Genes regularly present in pathogenic S. pneumoniae in contrast to related commensal species

Locus no.^*a*^	Annotation and comment(s)^*b*^	Presence (%) in:			
		S. pneumoniae	S. pseudopneumoniae	S. mitis	S. oralis
SP_0041	Bacteriocin BlpO (BlpU); downregulated in CSF (14)	100	100	0	0
**SP_0082**	Pneumococcal adhesion and virulence protein B, Pav; significantly upregulated in the heart (86)	92	100	5	0
**SP_0088-9**	Hypothetical protein; upregulated in CSF (14)	85	0	0	0
**SP_0102**	Glycosyl transferase	92	0	15	0
SP_0109	Putative bacteriocin	100	0	15	12
SP_0116	Hypothetical protein	100	67	0	0
**SP_0117**	Pneumococcal surface protein A (PspA); interferes with complement activation and binds lactoferrin; significantly upregulated in blood, CSF, the heart, and epithelial contact (14, 86)	100	0	0	0
SP_0124-5	Class IIb bacteriocins, CibA and CibB	100	100	10	0
**SP_0133-9**	Glycosyl transferase, glycosyl transferase, family 2, ABC transporter, ATP-binding protein, GlcNAc-PI de-*N*-acetylase, 3× hypothetical proteins	100	0	0	0
**SP_0175-8**	Riboflavin biosynthesis (RibD, RibE, RibA, RibC/H); essential for intracellular survival of *Brucella abortis* (96)	100	100	5	6
SP_0181	Conserved hypothetical protein	100	0	0	35
**SP_0245-53**	Putative pyruvate formate-lyase-activating enzyme, transcriptional regulator DeoR family, transcriptional regulator, PTS, putative formate acetyltransferase, transaldolase family protein, glycerol dehydrogenase (GldA)	100	0	20	0
**SP_0314-5, SP_0317-30**	Hyaluronate lyase (Hyl) (P, H), hypothetical protein, 4-hydroxy-2-oxoglutarate aldolase, carbohydrate kinase (PfkB family), conserved domain protein, oxidoreductase (H), hyaluronate PTS transporter (L), glucuronyl hydrolase (L), preprotein translocase (YajC subunit), oligohyaluronate lyase/heparinase II/III-like protein, hypothetical protein, sugar binding transcriptional regulator (RegR) (repression of glycuronidase expression), two transposases; colonization, meningitis	100	0	0	18
SP_0379	MFS (major facilitator superfamily) permease (L); import or export of target substrates	85	100	5	0
SP_0389	Hypothetical protein/cell wall-binding repeat protein	92	0	5	0
**SP_0390**	Choline-binding protein G (CbpG); upregulated in blood (14)	100	33	0	6
**SP_0394-7**	Mannitol PTS, transcriptional regulator	85	100	0	0
SP_0448	Hypothetical protein	100	0	0	0
SP_0449	Hypothetical protein	100	100	15	0
SP_0455	Hypothetical protein	92	0	0	0
SP_0471-2	Conserved hypothetical proteins	100	0	5	0
**SP_0473-8**	Putative regulator, PTS, lactose specific, GH20_hexosaminidase superfamily domain protein, 6-phospho-β-galactosidase	85	0	5	0
**SP_0504-10**	Type I restriction-modification system, M/R subunits (HsdM, HsdR); regulates capsular polysaccharide expression (32); SP_504 is missing in SPPN	100	100	0	0
SP_0528	Peptide pheromone BlpC	100	100	20	12
SP_0529	Bacteriocin secretion accessory protein BlpC, ABC transporter	85	100	20	12
SP_0576	Transcription antiterminator LicT	92	100	5	0
SP_0577-8	Beta-glucoside PTS transporter (BglP), 6-phospho-beta-glucosidase (BglA-2)	92	100	0	0
**SP_0582**	Endonuclease; significantly downregulated in CSF (14)	92	100	0	0
**SP_0595-6**	Hypothetical protein	92	0	0	0
**SP_0633**	Hypothetical protein	85	67	0	0
SP_0684-5	Bacteriocin, lactococcin 972 family protein	92	100	10	12
SP_0703-7	Peptide ABC transporter; downregulated in blood (14)	100	0	15	6
SP_0772	Hypothetical protein	100	0	0	0
SP_0858-60	Two membrane proteins, pyrrolidone-carboxylate peptidase (Pcp-1)	100	100	0	0
**SP_0886-92**	Type I restriction-modification system	100	100	0	54
SP_0899	Conserved hypothetical protein	92	100	10	47
SP_0950	Acetyltransferase, GNAT family	85	0	0	0
SP_0994	Hypothetical protein	100	100	0	0
SP_0997-8	Hypothetical protein (SP_0997-8 constitute one ORF)	100	100	0	0
**SP_1032-5**	Iron compound ABC transporter (Pit)	100	0	0	0
**SP_1038-40**	Phage-associated proteins, including site-specific resolvase	100	0	0	6
SP_1047-8	Hypothetical protein (part of RD6); in S. mitis B6 and SK637, the two constitute a single ORF	92	0	10	0
SP_1108	Hypothetical protein (fragment of SK142_0963)	92	0	0	0
SP_1210	Putative bacteriocin immunity protein	85	100	0	0
SP_1223-4	Toxin-antitoxin module	100	100	20	0
SP_1237	Acetyltransferase, GNAT family	100	100	10	18
SP_1250	McrBC 5-methylcytosine restriction system component	100	33	15	12
SP_1251	Putative endonuclease/restriction enzyme B	92	0	10	24
SP_1264	Conserved domain protein	85	100	0	0
SP_1427	Peptidase, U32 family (fragment of SK142_1155)	100	0	0	0
**SP_1430-1**	Type II DNA restriction-modification system	92	100	0	0
**SP_1433-8**	Transcriptional regulator, AraC family, drug ABC transporter	85	0	0	0
SP_1493-5	Hypothetical proteins; SP_1494 is fragment (84 aa) of 1,256- to 2,522-aa collagen binding protein (LPXTG cell wall anchor domain protein) of S. mitis	100	0	15	0
**SP_1500**	Glutamine ABC transporter (GatB), amino acid-binding protein; adhesin upregulated in contact with lung epithelia (13); remaining part of transporter (SP_1501-2) is present in all strains	100	100	10	24
**SP_1550**	Glutathione *S*-transferase family protein; upregulated in CSF (14)	100	0	20	0
SP_1656	Hypothetical protein	92	0	0	0
SP_1658	Membrane protein	92	0	15	0
SP_1679	Hypothetical protein	100	100	10	0
SP_1684	PTS, IIBC components	92	100	5	0
SP_1694	Acetyl xylan esterase	100	100	0	0
**SP_1704-6**	ABC transporter, unknown function	100	100	15	0
SP_1795	Beta-fructosidase; pneumonia	100	100	5	0
SP_1809-10	Transcriptional regulator, hypothetical protein; tryptophan biosynthesis	85	100	5	0
SP_1821	Sugar-binding transcriptional regulator, LacI family	100	100	20	12
SP_1822	Hypothetical protein	85	67	10	0
**SP_1823**	MgtC/SapB family protein/magnesium transporter	100	100	15	12
**SP_1824-34**	Phosphate ABC transporter, hypothetical protein, UDP-glucose 4-epimerase (GalE-2), galactose-1-phosphate uridylyltransferase (GalT-1), PhoU family transcriptional regulator, two hypothetical proteins, cell wall-anchored protein, cell wall surface anchor family protein (SP_1828-30 constitute RD11)	100	100	20	0
**SP_1856**	Transcriptional regulator, MerR family, galactose operon	100	100	15	24
**SP_1859**	Nicotinamide mononucleotide transporter PnuC	100	100	20	71
SP_1883-5	Putative dextran glucosidase DexS/alpha-phosphotrehalase/alpha-amylase, trehalose PTS system, IIABC components, trehalose operon transcriptional repressor (TreR)	100	100	10	35
SP_1894	Sucrose phosphorylase (GtfA)	100	0	10	76
**SP_1895-7**	Raffinose ABC transporter (RafG, RafF, RafE); upregulated in contact with lung epithelia (13)	100	0	0	0
**SP_1898-9**	Alpha-galactosidase (Aga), *msm* operon regulatory protein (MsmR)	100	0	10	76
SP_1917	Hypothetical protein	100	0	0	12
SP_1918	ABC transporter, ATP-binding protein (fragment of SK142_1598)	100	0	0	0
**SP_1923-6**	Pneumolysin (Ply), significantly upregulated in the heart (86); downregulated in CSF and by epithelial contact (14); hypothetical proteins	100	100	15	0
SP_1930-3	Hypothetical proteins	100	0	5	0
SP_1934-6	Hypothetical protein, Zn-dependent peptidase ImmA, M78 family (posttranslational modification, protein turnover), type II restriction-modification system regulatory protein	100	67	5	0
**SP_1937**	Autolysin (LytA) significantly upregulated in the heart (86); downregulated in CSF (14)	100	100	20	0
SP_1946	Putative transcriptional regulator PlcR	92	0	10	0
SP_1958	Hypothetical protein	100	0	5	0
SP_1989	Transcriptional regulator PlcR	92	0	10	0
SP_1992	Cell wall surface anchor family protein (“Xisco”)	100	0	0	0
**SP_2016-24**	Nicotinate-nucleotide pyrophosphorylase (NadC); downregulated in CSF (14); dicarboxylate carrier protein; downregulated in CSF (14), ABC transporter, ATP-binding protein (truncation), transcriptional regulator, GntR family, 6-phospho-beta-glucosidase; upregulated in CSF and by epithelial contact (14), PTS; upregulated in contact with lung epithelium (13), putative lactose/cellobiose transporter (SP_2018 is degenerate transposase)	100	0	0	0
SP_2081-3	Conserved hypothetical protein, two-component histidine transcriptional regulator PnpR/S (TCS04); activates the expression of the genes encoding the PstI transporter and PhoU1 regulator (SP_2084-8); genetic fitness, immune evasion, controls the expression of pneumococcal surface antigen (PsaA, SP_0117) (27)	100	100	0	0
**SP_2084-8**	Phosphate ABC transporter (Pst1: PstS, -C, -A, and -B proteins), phosphate system regulatory protein PhoU	100	100	0	0
**SP_2136**	Choline-binding protein PcpA; significantly upregulated in the heart (86)	100	33	5	6
SP_2148-51	Arginine deiminase pathway enzymes ArcA, ArgF, ArcC; orthologs in S. oralis subsp. *dentisani*	100	0	0	29
SP_2152-3	Conserved hypothetical protein, peptidase, M2i0/M25/M40 family	100	0	0	0
**SP_2158-68**	Fucose utilization operon including PTS (FucI, FucU, FucA); sepsis (64); upregulated in lungs versus nasopharynx (60); SP_2162 (*ptnC*); SP_2164 (PM0834) (mannose family PTS (SP_1858-66 constitute RD13)	85	0	10	0
**SP_2190/1**	Choline-binding protein A (PspC/CbpA)/isoprenylcysteine carboxyl methyltransferase; upregulated by epithelial contact (14); binds polymeric immunoglobulin receptor; binding of factor H minimizes the activity of the alternative pathway of complement and therefore enhances the capacity of the isolate to evade opsonophagocytosis	100	0	0	0
SP_2192-3	Two-component histidine transcriptional regulator (TCS06); regulates CbpA (SP_2190) expression (27); present in strains of S. oralis subsp. *tigurinus* in the context of isoprenylcysteine carboxyl methyltransferase (ICMT) family protein and IgA-binding beta-antigen genes	100	0	0	29

a Locus numbers in boldface refer to genes identified as associated with attenuated virulence in mouse models of pneumonia and/or bacteremia in one or more of three STM studies (10–12).

b CSF, cerebrospinal fluid; ORF, open reading frame.

### Cell wall teichoic acid biosynthesis.

Denapaite and coworkers (38) identified 18 genes that are involved in the synthesis of cell wall teichoic acids in S. pneumoniae and present also in S. mitis strain B6, except for the replacement of a glycosyltransferase gene (Spr0091 [SP_0102]) with another glycosyltransferase gene of family 2 (amylovoran biosynthesis protein). The latter substitution was also found in S. pneumoniae serotype 5 strain 70585 (38), in accordance with the special teichoic acid structure in that serotype (39).

Examination of the genomes of this study confirmed the findings of Denapaite et al. (38) concerning S. pneumoniae. Out of the 20 S. mitis strains, three (SK137, SK608, and SK1126) were identical to strains of S. pneumoniae and *S. pseudopneumoniae*, with the possible exception of SK674, in which no gene equivalent of SP_0102 was identified. The remaining 17 S. mitis strains were identical to S. mitis B6 and S. pneumoniae 70585, i.e., substitution of the equivalent of SP_0102. Like the majority of S. mitis strains, all strains of the three subspecies of S. oralis and all strains of *S. infantis* possessed the equivalent of the S. pneumoniae 70585 gene SP70585_RS00795. Thus, S. pneumoniae 70585 serotype 5 is exceptional among the 13 pneumococcal strains and identical to the majority of S. mitis strains and all strains of S. oralis and *S. infantis* in this respect. While the Spr_0091-encoded glycosyltransferase is proposed to catalyze the attachment of *N*-acetylgalactosamine (GalNac) or glucose to the teichoic acid repeating unit, the substitution gene in 70585 and the majority of S. mitis strains and all strains of S. oralis and *S. infantis* apparently incorporate a galactose unit instead, resulting in the teichoic acid intermediate Gal-AATGal-PP-upr (38).

We previously reported the structure and immunochemical properties of wall teichoic acid in S. mitis SK137, which is identical to the C polysaccharide of S. pneumoniae strains (40). This is supported by the genetic analysis presented in Table S3. The same blocks of genes involved in teichoic acid biosynthesis and transport were present in S. mitis strains SK137, SK608, and SK1126 and in all three strains of *S. pseudopneumoniae*. However, the remaining strains of S. mitis appear to share teichoic acid structure with S. pneumoniae serotype 5 (strain 70685), a difference that was not detected by two monoclonal antibodies directed against the teichoic acid backbone and phosphorylcholine, respectively (40).

Three S. mitis strains, SK564, SK575, and SK578, lacked homologs of the genes SP_1273, SP_1274, SP_1364, SP_1365, and SP_1366, like all strains of *S. infantis* and S. oralis. This is in agreement with our previous observation that the S. mitis and S. oralis strains did not react with a monoclonal antibody against the backbone of the pneumococcal C polysaccharide but reacted with an antibody against phosphorylcholine (40). An additional strain of S. mitis, SK1073, lacked three of these genes: SP_1364, SP_1365, and SP_1366. This suggests more structural differences within S. mitis than previously anticipated (40). An exception from these patterns are two strains of S. oralis subsp. *tigurinus*, Az-3a and SK255. They possessed all of the five mentioned genes but all as part of the third locus of teichoic acid biosynthesis genes (Table S3).

Homologs of Sor_0761, Sor_0762, and Sor_0763, which are suggested to add GalNac and phosphorylcholine to the repeating unit of S. oralis teichoic acid based on an examination of S. oralis Uo5 (31), were identified in all six strains of S. oralis subsp. *oralis.* However, they were absent from strains of S. oralis subsp. *tigurinus* and subsp. *dentisani*, suggesting structural differences of wall teichoic acid among the three subspecies of S. oralis (Table S3). Thus, teichoic acid of subsp. *tigurinus* and *dentisanii* appear to lack phosphorylcholine, with the striking exception of the two strains AZ_3a and SK255.

### Hyaluronidase.

One of the operons present in S. pneumoniae but not in S. mitis encompasses 16 genes, which include the hyaluronate lyase gene. In addition to all 13 S. pneumoniae strains, three S. oralis subsp. *tigurinus* strains, AZ_3a, SK313, and SK1074, contained the entire cluster of 16 genes flanked by orthologs of SP_0313 (glutathione peroxidase) and SP_0332 (*S*-adenosyl-methyltransferase MraW) in TIGR4. The operon included genes encoding hyaluronate lyase, 4-hydroxy-2-oxoglutarate aldolase/2-dehydro-3-deoxyphosphogluconate aldolase, carbohydrate kinase (PfkB family), putative sugar-phosphate isomerase, a putative gluconate 5-dehydrogenase, components IIA to IID of an associated PTS, glucuronyl hydrolase, preprotein translocase-YajC subunit, oligohyaluronate lyase/heparinase II/III-like protein, sugar binding transcriptional regulator RegR (repression of glucuronidase expression), and two transposases. One of these transposases interrupts the oligohyaluronate lyase/heparinase II/III-like protein gene in TIGR4. The entire block of genes corresponding to SP_0314 to SP_0334 was missing from strains of S. mitis, *S. pseudopneumoniae*, *S. infantis*, S. oralis subsp. *oralis*, S. oralis subsp. *dentisani*, and the two remaining S. oralis subsp. *tigurinus* strains, SK255 and SK304.

### Pneumolysin and autolysin.

Highly conserved pneumolysin genes were identified in all strains of S. pneumoniae and *S. pseudopneumoniae* and in three of the 20 strains of S. mitis, SK564, SK597, and SK1080. Likewise, highly conserved autolysin genes were identified in the same strains and, in addition, in S. mitis B6. While the autolysin gene in S. pneumoniae strains is 957 bp, that of the orthologous gene in *S. pseudopneumoniae* and in the three S. mitis strains SK564, SK597, and SK1080 was 951 bp, in accordance with previous observations (41, 42). The exception was S. mitis B6, which had the 957-bp allele as described by Romero et al. (43). The three S. mitis strains that possessed both pneumolysin and autolysin genes lacked the approximately 5,500-bp sequence that separates the two genes in S. pneumoniae and *S. pseudopneumoniae*, including the IS*1381* transposases OrfA and OrfB (SP_1927-SP_1928) and seven hypothetical proteins of phage origin (Fig. S3A).

10.1128/mBio.01985-19.3 FIG S3 (A) Highly conserved pneumolysin and autolysin genes in all strains of S. pneumoniae (represented by TIGR4) and *S. pseudopneumoniae* and in three of 20 strains of S. mitis (represented by SK597 and SK1080). The three S. mitis strains that possessed both pneumolysin and autolysin genes lacked the approximately 5,500-bp stretch that separates the two genes in S. pneumoniae and *S. pseudopneumoniae*, including the IS*1381* transposases OrfA and OrfB (SP_1927-8) and seven hypothetical proteins of phage origin. (B) Bacteriocin loci in S. pneumoniae and related commensal streptococci. Blp bacteriocin cassette with various numbers of bacteriocin and immunity genes, in addition to genes encoding a CAAX amino-terminal protease, PncP (SP_0547), a three-component response regulator (*blpR* [SP_0526] in TIGR4, *blpH* [SP_0527], and peptide pheromone *blpC* [SP_0528]) and one or two ABC transporters. One of the latter was degenerated in eight of the pneumococcus strains, including TIGR4. All strains of S. mitis and *S. pseudopneumoniae* had a structurally similar cassette located in the same context in the genome and containing the response-regulator genes. (C) Bacteriocin loci in S. pneumoniae and related commensal streptococci. Shown is the pneumocyclicin system cassette consisting of six genes present in three of the 13 S. pneumoniae strains and in all three strains of *S. pseudopneumoniae*. In the *S. pseudopneumoniae* strains, several bacteriocin and immunity protein genes in addition to three choline-binding protein genes and three ethanolamine utilization genes supplemented the cassette. S. mitis strains and strains of S. pneumoniae without the pneumocyclicin cassette had a locus in the same spot with variable numbers of bacteriocin and immunity protein genes. Download FIG S3, PDF file, 1.0 MB. Copyright © 2019 Kilian and Tettelin. 2019 Kilian and Tettelin This content is distributed under the terms of the Creative Commons Attribution 4.0 International license.

A 957-bp paralogous autolysin gene, presumably of phage origin and located immediately downstream of phage holin and antiholin genes, was present in genomes of five of the S. pneumoniae strains, P1031 (SPP_RS00395), 670 (SP670_RS00485), JJA (SPJ_RS09160), Hungary19A (SPH_RS00315), and 70585 (SP70585_RS00390), and in the three *S. pseudopneumoniae* strains in a region that is syntenic in the five pneumococcal strains. In addition, ATCC 700669 contained a paralog of this autolysin gene (SPN23F_15290) but in a different location. The three *S. pseudopneumoniae* strains had an additional allele of an autolysin gene located in another part of the genome. Five additional strains possessed one or more genes annotated as autolysin and being 867 to 1,011 bp in size, i.e., S. mitis SK137 (SK137_1963-1964 and SK137_0220), the two S. oralis subsp. *tigurinus* strains AZ_3a (H354_06746) and SK313 (HMPREF9950_1978), S. oralis subsp. *oralis* SK143 (SK143_ 0882 and SK143_1649), and S. oralis subsp. *dentisani* F0392 (HMPREF9178_1047).

### Sialidases.

Pneumococci encode up to three sialidases, NanA, NanB, and NanC, that have been identified as virulence factors (44, 45). NanA is a promiscuous exo-α-neuraminidase that hydrolyzes the removal of terminal α-(2→3)-, α-(2→6)-, and α-(2→8)-linked *N*-acetylneuraminic acid (Neu5Ac) residues from a variety of glycoconjugates. NanB is an intramolecular *trans*-sialidase producing 2,7-anhydro-Neu5Ac selectively from R2,3-sialosides. NanC also has strict α-(2→3)-neuraminidase activity, and the ﬁrst product is 2-deoxy-2,3-didehydro-*N*-acetylneuraminic acid (Neu5Ac2en), which can be slowly hydrated by the enzyme to *N*-acetylneuraminic acid. It was proposed that by adopting distinct catalytic mechanisms, NanA, NanB, and NanC work together to coordinate the sialidase action associated with pneumococcal virulence (45). Five of the pneumococcal strains had all three genes, as revealed by phylogenetic analysis with previously identified genes as references (Fig. S4) (note that many sequences in databases are mislabeled). The exceptions (encoding genes missing) were NanB in Hungary19A and NanC in D39, R6, Taiwan19F, 70585, and TCH8431.

The phylogenetic analysis demonstrated that NanA, in addition to S. pneumoniae, was encoded by 11 of 20 S. mitis strains, by all strains of S. oralis subsp. *oralis*, by three of five S. oralis subsp. *tigurinus* strains, and by four of six *S. infantis* strains but not by any of the *S. pseudopneumoniae* and S. oralis subsp. *dentisani* strains. NanB was found in six of 20 S. mitis strains and in one of the three *S. pseudopneumoniae* strains but in none of the strains of S. oralis and *S. infantis*. NanC was found exclusively in the seven mentioned S. pneumoniae strains. The phylogenetic analysis revealed at least two additional clusters of putative sialidases with LPXTG cell wall anchor motifs (Fig. S4). These unnamed putative sialidases were present in all three strains of *S. pseudopneumoniae* and in selected strains of S. mitis, the individual strains having two distinct genes (Fig. S4).

10.1128/mBio.01985-19.4 FIG S4 Phylogenetic analysis of neuraminidase/sialidase genes disclosing their presence in the individual taxa. In addition to *nanA*, *nanB*, and *nanC*, the analysis disclosed at least two additional clusters of putative sialidases with LPXTG cell wall anchor motifs. These unnamed putative sialidases were present in all three strains of *S. pseudopneumoniae* and in selected strains of S. mitis, the individual strains having two distinct genes. The figures at individual nodes represent bootstrap values. Download FIG S4, PDF file, 0.2 MB. Copyright © 2019 Kilian and Tettelin. 2019 Kilian and Tettelin This content is distributed under the terms of the Creative Commons Attribution 4.0 International license.

### Zinc-metalloprotease genes.

**FIG 3:**
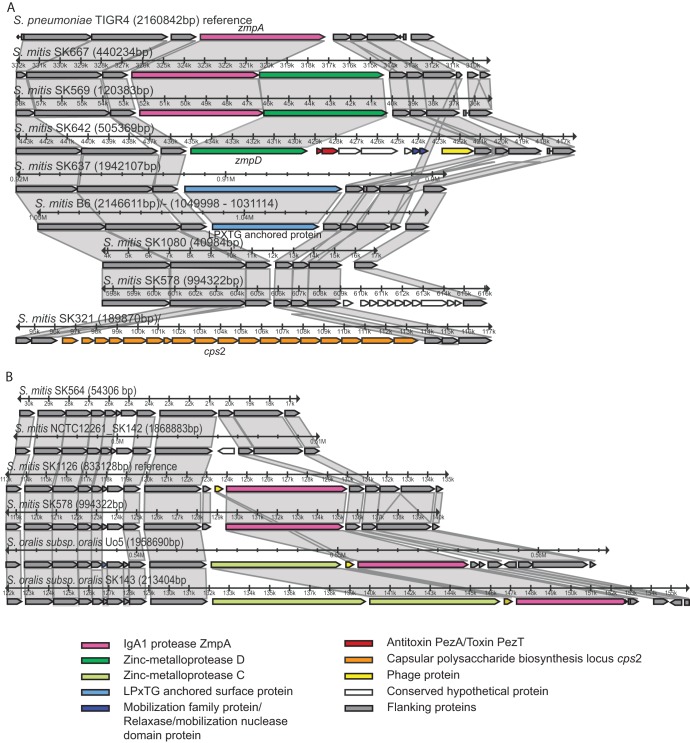
Comparisons of the IgA1 protease/*zmpA* loci in representative strains of S. mitis demonstrate that the locus is a hot spot for recombination. (A) Cluster II strains of S. mitis (represented by SK667 and SK569) show the same structure as pneumococci (represented by TIGR4). In some strains, the locus includes the paralogous *zmpD* gene. Strains of S. mitis cluster I lack the *zmpA* gene at this site, but some strains instead have a gene encoding an unrelated LPxTG-anchored protein (SK637 and B6) or a complete capsular polysaccharide biosynthesis locus (SK321). (B) Some strains of S. mitis cluster I carry the *zmpA* gene in the same spot as S. oralis but without the paralogous *zmpC* gene.

The phylogenetic analysis of genes combined with results of the *in vitro* assay for IgA1-protease activity revealed that the *zmpA* (*iga*) gene was present in all strains of S. pneumoniae and *S. pseudopneumoniae*, in 11 of 20 strains of S. mitis, and in all strains of S. oralis subsp. *oralis* but not in any strain of S. oralis subsp. *tigurinus* and *dentisani* or *S. infantis* (although IgA1 protease activity may be found in some strains of *S. infantis* [46]). The location of the *zmpA* gene in positive strains showed a striking dichotomy. In all strains of S. pneumoniae, *S. pseudopneumoniae*, and the eight S. mitis strains of cluster II (Fig. 1B), the *zmpA* gene was flanked upstream by genes encoding exonuclease RexA/ATP-dependent helicase and a hypothetical protein (corresponding to SP_1152 and SP_1153 in TIGR4). The second pattern was seen in two S. mitis cluster I strains (SK578 and SK1126). In these strains, the *zmpA* gene (SK578_0762 and SK1126_0642) was present in a region syntenic to the *zmpA* gene region in S. oralis subsp. *oralis* strains, S. oralis genomosubspecies 1 ATCC 6249, and in *Streptococcus* sp. strain SK643, apart from the fact the S. oralis strains had two or three paralogous *zmp* genes in the locus (Fig. 3). The S. mitis cluster II strains with the *zmpA* locus identical to that of S. pneumoniae show synteny to this region but without the additional *zmp* genes. This suggests that the *zmpA* gene was reacquired in strains SK578 and SK1126 by homologous recombination with a strain of S. oralis subsp. *oralis*. This conclusion is supported by the close genetic relationship of the *zmpA* gene of SK578, SK1126, and SK643 with *zmpA* genes from S. oralis subsp. *oralis* strains rather than with those of other strains of S. mitis (not shown).

The *zmpA* locus appears to be a hot spot for recombination, as evidenced by several observed modifications. Among the 13 S. pneumoniae strains, six strains (TIGR4, Taiwan19F, TCH8431, R6, D39, and 70585) showed the exact same pattern as TIGR4. In strains G54, CGSP14, ATCC 700669, and JJA, like in six out of 20 S. mitis strains, the paralogous *zmpD* gene was found immediately downstream of *zmpA*, in strain G54 followed by a transposase gene. In the three remaining pneumococcal strains, P1031, 670, and Hungary19A, a large insert was seen downstream of the *zmp* gene(s) (*zmpA* and *zmpD*). The insert in P1031 was approximately 53 kb and in the two other strains approximately 33 kb, and it included multiple genes reminiscent of a phage. Part of the insert was inverted in 670 and Hungary19A relative to its orientation in P1031. S. mitis strain SK1073 showed the same structure as S. pneumoniae P1031. Strains of S. oralis subsp. *oralis* show synteny with at least 30 kb surrounding the *zmpA* region in S. pneumoniae TIGR4 but without the *zmpA* gene.

In strains of S. mitis missing the *zmpA* gene, a variety of other genes were found in the locus. Strain SK642 had *zmpD* and several other genes (SK642_940-947) in the locus but no *zmpA* gene, in agreement with lack of IgA1 protease activity. As we recently reported, two out of 20 S. mitis strains examined for capsular biosynthesis locus (*cps*) genes, SK321 and SK137, possessed a second *cps* operon (*cps2*) in this locus (16). S. mitis strains B6, SK1080, and SK637 have, in the same locus, genes (smi_1064 in B6, HMPREF9957_1097 in SK1080, and Sm_SK637_00942) encoding another LPXTG cell wall anchor domain protein of 1,702, 1,256, and 2,522 amino acids (aa), respectively, with homology to proteins annotated as collagen adhesin/mucus binding protein in other strains of S. mitis. These proteins are unrelated to the IgA1 protease and have the LPXTG anchor in the C-terminal and not the atypical N-terminal location in IgA1 proteases.

In several strains, the *zmpA* locus included phage proteins and multiple hypothetical proteins presumably of phage origin, mobilization protein and relaxase systems known to be involved in conjugative mobilization, and toxin-antitoxin systems (Fig. 3), which may contribute to the striking variability of the locus.

All strains of the species S. pneumoniae, S. mitis, and *S. pseudopneumoniae* possessed a *zmpB* gene in the same context of the genome. In addition, two pneumococcal strains (TIGR4 and G54) and five S. mitis strains (NCTC12261, SK608, SK637, SK667, and SK1126) possessed a *zmpC* gene. In all strains of S. oralis subsp. *oralis*, one or two homologs of the *zmpC* gene was/were located immediately downstream of *zmpA*.

### Carbohydrate uptake systems.

The S. pneumoniae sugar transporters primarily consist of phosphoenolpyruvate-dependent phosphotransferase system (PTS) transporters and carbohydrate-specific ATP-binding cassette (ABC) transporters. A total of 32 carbohydrate uptake systems are encoded by the S. pneumoniae TIGR4 genome, 21 of them being PTS sugar-specific enzyme II complexes, eight ABC sugar uptake systems (seven CUT1 type and one CUT2 type), in addition to two facilitators, and one symporter, rendering the pneumococcus extremely versatile with regard to sugar utilization (33, 47). As shown in Table S1E, some variation was found among the 13 pneumococcus strains. Relative to TIGR4, 17 PTS, three ABC transporters of CUT1 type specific for *N*-acetylneuraminic acid (SP_1688-1690), sucrose (SP_1796-1798), alpha-galactosides (SP_1895-1897), and one symporter were absent from all or nearly all strains of S. mitis, S. oralis, and *S. infantis* (data not shown). Most of these were also missing from *S. pseudopneumoniae* (Table S1E). According to the demonstrated sugar specificity of the transporters (47), the uptake systems missing from the three mutualistic species will affect their ability to take up α- and β-glucosides, α-galactosides, pentoses, mannitol, fucose, l-arabinose, threhalose, ascorbate, sulfated glycosaminoglycans (part of the RegR regulon/hyaluronidase locus), and *N*-acetylglucosamine, taking into account that some systems are duplicated. The ability of S. pneumoniae strains to hydrolyze esculin and to ferment inulin, glycogen, mannitol, threhalose, and *N*-acetylgalactosamine, in contrast to the other species, is in agreement with this (48, 49). No sugar-specific PTS unique to S. mitis strains was identified except for distinct sorbose-specific PTS-Fru systems at different locations in SK608 (SK608_503-506 [subunits IIA to D]), SK642 [SK642_372-375 (subunits IIA to D]), and SK597 [SK597_RS04410-RS04425 (subunits IIA to D]), and a PTS sugar-specific permease component in SK637 (SK637_00220).

### Other ABC transporters.

S. pneumoniae contains a high number of ATP-dependent transporters (33). In addition to the three CUT1 type ABC carbohydrate transporters, seven additional pneumococcal ABC transporters were absent from the mutualistic species S. mitis, S. oralis, and *S. infantis* (Table S1F). They included two phosphate transporters, SP_1824-1826 and SP_2084-2086, present in S. pneumoniae and *S. pseudopneumoniae* strains, both part of larger blocks of 16 (SP_1821-1836) and 8 genes (SP_2081-2088), respectively, absent from the species S. mitis, S. oralis, and *S. infantis* (Table S1F). In addition, an iron compound transporter system (SP_1032-1035) was unique to S. pneumoniae.

A peptide ABC transporter system, SP_0703-0707, was present in all S. pneumoniae strains, in three S. mitis strains (B6, SK608, and SK629), and in S. oralis subsp. *tigurinus* AZ_3a. A drug transporter, SP_1434-1435, was part of a block of six genes (SP_1433-1438) exclusive to 11 of the 13 S. pneumoniae strains. Finally, an ABC transporter, SP_1704-1706, of unknown function was present in all strains of S. pneumoniae and *S. pseudopneumoniae* and three strains of S. mitis, SK564, SK597, and SK608 (Table S1F).

### CBPs.

Choline-binding proteins (CBPs) are anchored to the cell wall by hydrophobic interactions with choline-containing teichoic acids (50, 51). They are composed of a choline-binding module consisting of repeats of 20 amino acids and a nonconserved functional domain. Among the 15 choline-binding protein genes that previously were detected in S. pneumoniae TIGR4 (33), four (in addition to *lytA*, described above) were almost unique to pneumococci. Choline-binding protein A (CbpA; SP_2190) was exclusively present in the 13 strains of S. pneumoniae. This was also the case for the two partially homologous versions of pneumococcal surface protein A (PspA; SP_0117).

Choline-binding protein G (CbpG; SP_0390) was present in all strains of S. pneumoniae, in one strain of *S. pseudopneumoniae* (IS7394), and in S. oralis subsp. *tigurinus* strain Az_3a. The *cbpG* genes in *S. pseudopneumoniae* IS7394 and S. oralis subsp. *tigurinus* strain Az_3a were 98% identical (on the nucleotide level) and approximately 400 nt longer than the orthologous genes in S. pneumoniae strains. Choline-binding protein PcpA (SP_2136) was present in 12 of the 13 strains of S. pneumoniae (absent from P1031), in one strain of *S. pseudopneumoniae* (IS7394), in S. oralis subsp. *tigurinus* strain Az_3a, and in S. mitis SK564.

S. mitis B6 was unique among the S. mitis strains in harboring a large number of additional CBP genes, many of which have unusual repeat domains with a 40mer repeat motif, as also noted by Denapaite et al. (24). One gene (B6:smi_0086), encoding a protein of 395 to 400 amino acids, was also present in 17 of the 20 S. mitis strains. The gene product in these strains was previously annotated as either CbpI, CbpC, CbpJ, choline-binding A domain protein, or cysteine-rich secretory family protein. In two S. pneumoniae strains, TIGR4 and G54, homologous sequences in the same locus were present as fragments (SP_0067-0069 and SPG_RS00355-RS00360). In other pneumococcus strains, a glyoxalase gene replaced the gene. Many of the remaining CBP genes present in B6 also were detected in one to five strains of S. mitis without association with their phylogenetic relationships (see below). In three of them, the functional domain was annotated as endo-beta-*N*-acetylglucosaminidase.

Uniquely among the S. oralis strains, the genome of S. oralis subsp. *tigurinus* encoded five CBPs, in agreement with its assumed expression of cell wall teichoic acid with choline. Surprisingly, this was not the case for SK255 with the same exceptional set of teichoic acid biosynthesis genes (Table S3).

### Proteins with LPxTG anchor.

The genome of S. pneumoniae TIGR4 contains 19 genes that encode proteins with an LPxTG motif, including the zinc-metalloproteases ZmpA, ZmpB, and ZmpC and the hyaluronidase (33). The largest protein expressed by S. pneumoniae TIGR4 is PsrP, a serine-rich surface glycoprotein with the characteristic repeats SASASA (SP_1772). A comparative genomic analysis of 42 invasive and 30 noninvasive clinical isolates of S. pneumoniae demonstrated that the presence of PsrP and associated proteins responsible for glycosylation and export correlated positively with the ability of S. pneumoniae to cause human invasive pneumococcal disease (52). The glycoprotein was shown to interact with keratin (52). Among the pneumococci examined, the locus was found in S. pneumoniae strains TIGR4 (SP_1772; 4,776 aa), Hungary19A (SPH_1885; 4,765 aa), ATCC 70669 (SPN23F17820; 4,433 aa), 70585 (SP70585_1816; 2,215 aa), JJA (SPJ_RS08290), and CGSP14 (SPCG_1750; 4,695 aa). A homologous protein, albeit with fewer SASASA motifs, and associated glycosylation genes were found in the commensal S. mitis strains B6 (smi_1662_1591; 5,322 aa), SK608 (SK608_0285; 3,821 aa), and SK642 (SK642_1513; 3,277 aa), in S. oralis subsp. *oralis* strains ATCC 35037 (HMPREF8579_0708; 5,322 aa); SK143 (SK143_1435; 2,075 aa), Uo5 (SOR_1583; 1,962 aa), and in S. oralis subsp. *tigurinus* SK313 (HMPREF9950_1398; 1,610 aa). This genomic islet occurs also in S. gordonii, where the homologue of the protein named PsrP, GspB, has been associated with endocarditis (53).

A gene of up to 11,001 bp that encodes an LPXTG cell wall anchor domain protein was detected immediately downstream of the *zmpB* gene in S. mitis strains NCTC 12261 (SK12261_1285), SK137, SK271, SK321, and SK1073. The gene was restricted to S. mitis strains of cluster I (see below) and encodes a repeat protein (DUF1542) unrelated to ZmpB.

### Pilus proteins.

The genome of five of the 13 pneumococcus strains included the pilus PI-1 island of seven genes (*rlrA*, *rrgA-C*, and *srtB-D*). None of the commensal streptococcus strains had homologs of these genes.

PI-2 pilus-encoding genetic islets initially discovered in some S. pneumoniae strains (54) were subsequently identified in strains of S. oralis (GenBank accession number JF496566.1), S. mitis, and S. sanguinis by Zahner et al. (55). Among our 13 S. pneumoniae strains, Taiwan19F and TCH8431_19A contained the five genes of the PI-2 pilus-encoding genetic islets (PitA, signal peptidase I [SipA], PitB, and two sortases, SrtG1 and SrtG2) flanked by ferrochelatase (SOR_RS05190) and peptidase T genes (SOR_RS05220), while none of the 20 S. mitis or three *S. pseudopneumoniae* strains did so. The five genes in the same genomic context were found in all six strains of S. oralis subsp. *oralis* and in three out of five strains of S. oralis subsp. *tigurinus* strains (AZ_3a, SK313, and SK1074) but not in S. oralis subsp. *tigurinus* SK255 and SK304 or in any of the five strains of S. oralis subsp. *dentisani*.

### Toxin-antitoxin systems.

The toxin-antitoxin module genes SP_1223-SP_1224, which are essential for bacterial persistence under stressful conditions, such as nutrient deprivation, antibiotic treatment, and immune system attacks (56), were present in all strains of S. pneumoniae and *S. pseudopneumoniae* but in only four of the 20 S. mitis strains: NCTC12261, SK271, SK1073, and SK642.

### Histidine triad proteins.

Members of the histone triad protein superfamily contain a repeated sequence region that includes a His-X-X-His-X-His (histidine triad) motif and are involved in Zn^2+^ homeostasis. Family member PhpA has been shown in vaccine studies to be a protective antigen in mice (57), and PhtD is being tested in humans as part of a pneumococcal vaccine (58). Five histidine triad protein genes are present in the TIGR4 genome (SP_0628, SP_1003 [*phtD*], SP_1004 [*phtE*], SP_1174 [*phpA*], and SP_1175 [*phtA*]) but in only five to eight other pneumococcal strains. One or two homologs (SP_0628 and SP_1074, both of which are smaller proteins of unknown function) were present in both S. pneumoniae and commensal species in the same location.

### Bacteriocins.

In accordance with previous observations (59, 60), all S. pneumoniae strains possessed the Blp bacteriocin cassette with various numbers of bacteriocin and immunity genes, in addition to genes encoding a CAAX amino-terminal protease, *pncP* (SP_0547), a three-component response regulator (*blpR* [SP_0526] in TIGR4, *blpH* [SP_0527], and peptide pheromone *blpC* [SP_0528]) and one or two ABC transporters. One of the latter was degenerated in eight of the pneumococcus strains, including TIGR4. All strains of S. mitis and *S. pseudopneumoniae* had a structurally similar cassette located in the same context in the genome and containing the response regulator genes. In addition, both ABC transporters *blpB* and *blpA* were present and intact in four of the 20 S. mitis strains and in all three *S. pseudopneumoniae* strains. The three *S. pseudopneumoniae* strains had one to three bacteriocin genes, whereas only occasional strains of S. mitis had bacteriocin genes in the cassette. None of the S. oralis strains possessed the cassette, with the notable exceptions of S. oralis subsp. *dentisani* strain 7746 and S. oralis subsp. *tigurinus* SK255, which had the same cassette genes as *S. pseudopneumoniae* strains supplemented by eight and five bacteriocin genes, respectively, and several immunity protein genes (Fig. S3B).

Bogaardt et al. (60) described a circular bacteriocin (pneumocyclicin) system encoded by a cassette of six genes upstream of the *comA* and *comB* genes. A recent report demonstrated that these genes are strongly upregulated during competence (61). This cassette was present in three of the 13 S. pneumoniae strains (70585, Hungary19F, and JJA) and in all three strains of *S. pseudopneumoniae*. In the *S. pseudopneumoniae* strains, several bacteriocin and immunity protein genes, in addition to three choline-binding protein genes and three ethanolamine utilization genes, supplemented the cassette. None of the strains of S. mitis, S. oralis, or *S. infantis* had the pneumocyclicin cassette, but S. mitis strains and strains of S. pneumoniae without the pneumocyclicin cassette instead had a locus in the same spot with various numbers of bacteriocin and immunity protein genes. Some S. mitis strains, in addition, had choline-binding protein genes similar to those of *S. pseudopneumoniae* (Fig. S3C).

The *cibABC* locus was present in all strains of S. pneumoniae and *S. pseudopneumoniae*. The gene encoding the immunity protein CibC also was present in all commensal strains. However, in accordance with a recent report (61), which showed absence from two S. mitis strains, *cibA* and *cibB* were absent from all other strains apart from S. mitis strains B6 and SK569.

Four other bacteriocins were present in all S. pneumoniae strains and, with few exceptions, absent from other strains: SP_0041 (present in three *S. pseudopneumoniae* strains), SP_0109, bacteriocin, lactococcin 972 family protein (present in IS7493, SK1073, SK1126, SK95, and Uo5), SP_0125, class IIb bacteriocin, lactobin A/cerein 7B family (present in SK569), SP_0684-85, bacteriocin, and lactococcin 972 family protein (present in all *S. pseudopneumoniae* strains, ATCC 6249, SK629, and SK642).

A search for bacteriocin genes with no orthologs in S. pneumoniae revealed a total of 19 genes among the commensals. These encoded ten class IIb bacteriocins/lactobin A, cerein 7B family peptides, eight class II bacteriocins with double-glycine leader peptides, and one unclassified bacteriocin, all with associated immunity protein genes. The individual bacteriocin genes were shared by two to eight strains of different species with no particular pattern.

### Prophages.

A recent study of nearly 500 pneumococcal genomes demonstrated that every pneumococcal genome contains prophage DNA. Although only some constitute full-length or putatively full-length prophages, 72% of them possess virulence genes (*pblA* and/or *pblB*), and transcriptomic data provide evidence of prophage gene expression (62, 63).

Our analysis revealed that four of the 13 S. pneumoniae genomes included complete prophages spanning from 40.2 to 94.4 kb. In addition, two of these plus two other pneumococcal genomes contained a putative phage, and all others contained incomplete phages spanning from 26.6 to 6.3 kb.

All 13 pneumococcus strains and S. oralis subsp. *tigurinus* strain AZ_3a had SP_1038, SP_1039, and SP_1040 homologs: two phage-associated proteins and a site-specific recombinase.

### Genes unique to commensal species.

A search for clusters of syntenic orthologs (29) that did not include proteins in the 13 S. pneumoniae strains revealed 210 proteins encoded by the genomes of 15 or more of the 47 strains of S. mitis, *S. pseudopneumoniae*, S. oralis, and *S. infantis*. Among these, 25 genes were present in 18 to all 20 of the S. mitis strains. Thirteen of these genes grouped in operons of up to four genes. They include three genes involved in 7-cyano-7-deazaguanine synthase/queuosine biosynthesis (SM12261_0489-91), the LrgAB genes involved in oxidative stress protection (SM12261_0495-6), four genes involved in hemin transport (SM12261_0576-9), and an ABC transporter system with associated 2-component histidine kinase regulator (B6:smi_1070-3). Interestingly, at least five genes are involved in oligopeptide binding, amino acid uptake, and synthesis of an essential amino acid, leucine (SM12261_0266, SM12261_0268, SM12261_1076, SM12261_1300, and SM12261_1517). The individual genes and their functions are listed in Table 2. In several cases, remnants of these genes were detectable in S. pneumoniae strains. Examples are a pseudogene of QueC consisting of the first 213 bp, a pseudogene of the transcriptional regulator, MarR family, associated with an ABC transporter, and a truncated version (N-terminal 360 bp) of the small subunit LeuD of the 3-isopropylmalate dehydratase heterodimer.

**TABLE 2:** Genes present in commensal *Streptococcus* species but absent from S. pneumoniae

Locus no. in S. mitis	Annotation and comments	Presence (%) in:				Size of protein (aa)
		S. mitis	*S. pseudopneum.*	S. oralis	*S. infantis*	
SM12261_0091-2	Gram-positive cell wall surface anchor family protein (YSIRK family); one reading frame in all other strains; N-terminal 199 nt show 87% identity, whereas remaining part shows no significant homology to S. pneumoniae	95	0	100	100	657
SM12261_0140	MarR family transcriptional regulator (phosphoenolpyruvate-protein phosphotransferase); absent from S. oralis subsp. *dentisani*; pseudogene in S. pneumoniae; regulators with the MarR-type HTH domain control a variety of biological functions, including resistance to multiple antibiotics, household disinfectants, organic solvents, oxidative stress agents, and regulation of the virulence factor synthesis in pathogens	100	0	65	0	81
SM12261_0266	Oligopeptide-binding protein SarA; present in four of five S. oralis subsp. *dentisani* isolates, ATCC 6249, and SK643	100	0	24	0	654
SM12261_0268	Amino acid permease 2 superfamily; APC (amino acid/polyamine/organocation) family permease (solute:cation symporter) involved in the uptake of a specific amino acid and/or polyamine substrate with the concomitant import of a proton	90	0	100	100	614
SM12261_0489-91	Queuosine biosynthesis proteins QueC, QueD, and QueE; queuosine is a hypermodified guanosine analog; pseudogene of QueC consisting of first 213 bp is present in S. pneumoniae strains	100	100	82	67	217, 147, 226
SM12261_0495-6	LrgA + LrgB; homologs of LrgA and LrgB/CidA and CidB in Streptococcus mutans and Staphylococcus aureus; substantial influence on properties involved in colonization and persistence in dental biofilms and under oxidative stress conditions (99, 100)	100	100	100	100	124–134, 231
SM12261_0576-9	Iron chelate uptake ABC transporter (FeCT family) (permease, ATP-binding protein, oligopeptide-binding protein) and putative esterase (predicted hydrolase of the alpha/beta superfamily)	100	0	100	0	348, 251, 336, 230
SM12261_0615	Beta-glucosidase 2, glycosyl-hydrolase family 116 N-terminal domain	100	100	100	83	102–127
SM12261_0623	Virulence factor BrkB; a homolog in Bordetella pertussis is essential for resistance to complement-dependent killing by serum	90	0	100	100	286–294
SM12261_1076	3-Isopropylmalate dehydratase, large subunit (LeuC); this enzyme performs the second step in the biosynthesis of leucine; the prokaryotic enzyme is a heterodimer composed of a large (LeuC) and small (LeuD) subunit; LeuD (591 nt) occurs downstream from LeuC; LeuD is present in strains of S. pneumoniae in a truncated version (N-terminal 360 bp) in an otherwise syntenic area	100	100	100	100	460
SM12261_1129	Glycerophosphoryl diester phosphodiesterase	100	0	0	0	587
SM12261_1229	PF08002 family protein of unknown function	95	0	59	50	153–181
SM12261_1300	Amino acid permease (LysP superfamily: l-asparagine transporter and related permeases)	100	0	100	100	445–470
SM12261_1517	Oligopeptide-binding protein AliD (SarA); located immediately upstream of the *cps* locus, in some strains flanked by the paralog *aliC*; pseudogene in association with *cps* loci of S. pneumoniae serotypes 25A, 25F, and 38 (104); SarA^−^ mutant in S. gordonii is defective in serum-induced aggregation, competence, growth on complex nitrogen sources, and ability to colonize the oral cavity (103)	95	0	100	0	652
SM12261_1764	Hypothetical protein; contains DNA polymerase III subunits gamma and tau domain; predicted coiled-coil domain-containing protein (DUF2360)	95	100	100	100	227–232
B6:smi_1070-3	ABC transporter, ATP-binding protein + ABC-2 transporter family protein + histidine kinase + response regulator receiver domain protein	95	100	71	33	302, 250, 292, 225

Several of the genes identified as being unique to the commensal streptococci were also missing from *S. pseudopneumoniae* (Table 2).

### Comprehensive search for species-specific genes.

To verify our predictions of species specificity of the 224 and 25 genes for S. pneumoniae and commensal species, respectively, we analyzed their presence in all available nucleotide sequence Multi-FASTA files of assemblies for genomes labeled S. pneumoniae (number of unique strains, 7,398), *S. pseudopneumoniae* (*n* = 43), S. mitis (*n* = 89), S. oralis (*n* = 75), and *S. infantis* (*n* = 11). The cutoff values used were 80% nucleotide sequence identity and 40% coverage. Results of this analysis adjusted for strain misidentifications detected by the phylogenetic analysis (Fig. S5A) confirmed that a majority of the 224 or 25 genes were specific to either S. pneumoniae or S. mitis, respectively (Table S4). Of the 224 S. pneumoniae-specific genes, 150 were present in at least 80% of the S. pneumoniae genomes analyzed and present in less than 20% of all S. mitis genomes. Most of the genes not matching these requirements encoded PTS or other transport systems, in accordance with known variations in carbohydrate utilization capabilities among pneumococci. Other notable exceptions are selected choline-binding proteins, including PspA (SP_0117). The latter is explained by the two distinct versions of *pspA*, of which only one was detected by BLASTN due to their overall low nucleotide identity. Three genes initially recorded as specific to S. pneumoniae (SP_1108, SP_1427, and SP_1918) had homologous sequences within larger genes in all S. mitis genomes located in the same genomic context. The functional implications are yet unknown.

10.1128/mBio.01985-19.5 FIG S5 (A) Midpoint-rooted circular tree based on >7,500 streptococcal genome assemblies. S. pneumoniae strain labels are colored in black, except for the 13 gap-free genomes that were selected for our detailed study that are shown in orange (*n* = 12) or magenta (TIGR4) and labeled with large orange or magenta circles. *S. pseudopneumoniae* labels are blue, S. mitis red, S. oralis green, and *S. infantis* cyan. Entire nonpneumococcal clades were further highlighted using the same colors. Taxa that were erroneously assigned to a given species are easily identified by mismatched colors within each of the clades, with the exception of the S. pneumoniae clade, which exclusively harbors S. pneumoniae strains. (B) SplitsTree phylogenetic analysis of 40 S. mitis genomes revealing multiple subclusters. Two genomes of S. pneumoniae were included in the analysis as references. Download FIG S5, PDF file, 2.1 MB. Copyright © 2019 Kilian and Tettelin. 2019 Kilian and Tettelin This content is distributed under the terms of the Creative Commons Attribution 4.0 International license.

Among the 25 commensal-specific genes, 24 were present in at least 80% of the S. mitis genomes analyzed and present in a minority of the 7,381 S. pneumoniae genomes. The exception was an amino acid permease gene, which was exclusive to S. mitis, S. oralis, and *S. infantis* but present in only 80 of the 104 S. mitis genomes.

### Association of S. mitis subclusters with origin of isolation and relevant genes.

To validate the observed subclustering of S. mitis (Fig. 1), we performed an alignment that included an additional 20 genomes of S. mitis with known origin that recently became available in the GenBank database. Whole-genome-level core single-nucleotide polymorphism (SNP)-based phylogenetic analysis of our 20 S. mitis genomes together with 20 publicly available S. mitis genomes, plus S. oralis Uo5 and S. pneumoniae TIGR4 and ATCC 700669 as outgroups, was performed. The analysis confirmed the initially observed clustering within the species. Based on the SplitsTree analysis shown in Fig. S5B and observed associations with genotypic findings, two major clusters, I and II, were recorded, each subdivided into three tentative subclusters. Table S5 shows the origin of the isolates and a summary of the presence of selected genes. As shown in the table, cluster I strains were distinct by having a nicotinamide biosynthesis operon and an acetyl-transferase gene, whereas cluster II strains were characterized by two distinct genes, annotated as platelet-aggregating factor Sm-hPAF (64)/fucolectin-related protein and both with a thiol-activated cytolysin domain, and a gene encoding a divalent cation transporter protein (Table S5). Strains of both clusters had a gene annotated as the early gene induced during competence induction, *comM* (55). The gene was present at the same location in all strains of S. pneumoniae and S. mitis but in S. mitis without the associated bacteriocin, immunity, and transporter genes. The *comM* gene in the two clusters of S. mitis was strikingly different, encoding proteins of only 40% amino acid identity. The allele in S. mitis cluster II strains was 95% identical (on a nucleotide level) to that of S. pneumoniae.

Neither the acetyltransferase gene nor one of the thiol-activating cytolysin genes (SK608_1252) was present in the S. pneumoniae strains. The other thiol-activating cytolysin gene (SK608_1273) was a pseudogene in all S. pneumoniae strains, in two of the *S. pseudopneumoniae* strains, and in three of the 12 strains of S. mitis cluster II strains (Table S5).

A previous study identified platelet-aggregating activity mediated by phage-associated proteins (phage tail proteins) PblA and PblB in a strain of S. mitis isolated from endocarditis (65). In the present collection of S. mitis strains, only SVGS_061 possessed these proteins.

All strains assigned to cluster II, except for strain SK1080, harbored the IgA1 protease gene *zmpA.* Interestingly, the gene and its enzyme activity in strains that were available for testing were also present in eight of the 26 S. mitis cluster I strains but in six of them at another location in the genome.

## DISCUSSION

Numerous focused studies have identified phenotypic properties of S. pneumoniae that are now generally recognized as crucial to virulence (3, 4, 9). In more comprehensive gene-based approaches, knockout mutants generated by signature-tagged mutagenesis (STM) were tested in mouse models of infections in the lungs and blood (10–12). These models detect genes essential for the ability of mutants to outgrow others in an inoculum pool of mutant strains. However, successful survival in competition with other members of the host microbiota also is important for mutualistic bacteria. Accordingly, many of the properties identified as virulence factors in S. pneumoniae also are present in genetically related commensal species (2, 16–19). Our study shows that 299 of 337 genes identified by STM as being attenuated for virulence in a mouse pneumonia model (12) were present in the commensal species S. mitis, S. oralis, and *S. infantis*. This includes the locus SP_2141-SP_2146, which is upregulated during pneumococcal contact with epithelial cells (14) and in which multiple STM hits by all three STM studies suggest that the region is important for the pathogenesis of pneumonia (10–12, 32). Likewise, this is the case for the vast majority of genes identified as being significantly upregulated in lungs and in blood (14, 66). Conceivably, these genes are crucial to successful survival of the bacteria in these niches but do not explain the differential pathogenic potential of pneumococci and the closely related species S. mitis. However, among eight pneumococcal virulence determinants with the highest levels of gene expression in the heart (15), which presumably reflects those required for disseminated infection, five were exclusive to or nearly exclusive to the pneumococci in this study: pneumococcal adhesion and virulence protein B (PavB; SP_0082), pneumococcal surface protein A (PspA; SP_0117), pneumolysin (Ply; SP_1923), autolysin (LytA; SP_1937), and pneumococcal choline-binding protein A (PcpA; SP_2136).

The foundation of our approach is an evolutionary model for S. pneumoniae and its close relative, S. mitis. We previously presented evidence to support the scenario that the two species share an immediate ancestor, a pneumococcus-like species presumably pathogenic to the immediate ancestor of hominids (18, 20). Members of this ancestral bacterial population faced a signiﬁcant selection pressure, presumably resulting from shortage of potential hosts, in two opposing ways. Lineages now recognized as the nonpathogenic S. mitis secured harmonious coexistence with their host by reductive evolution and stabilization of the genome (20). This process included partial loss of many recognized virulence factors, transposases, repeat elements, competence genes, and the Dpn restriction-modiﬁcation system, combined with acquisition or maintenance of CRISPR/Cas sequences (20). In contrast, the presumably single lineage now known as S. pneumoniae survived by optimizing its pathogenic potential and genomic and phenotypic plasticity secured by multiple regulatory mechanisms, frequent intraspecies gene exchange, and import of genes from S. mitis and other related viridans streptococci (20, 67, 68). This strategy proved successful with the more recent substantial expansion of the human population, as illustrated by the boost in the pneumococcal cluster of lineages generated by genetic diversification, mainly by horizontal DNA import (Fig. 1). (Note that the impact of the expansion of the host population on the numerous S. mitis lineages, which mainly spread vertically, is less clear, as it would require extensive sampling.) Building upon this foundation, detailed genome comparisons of representative collections of S. pneumoniae and S. mitis are likely to identify genes important for both pathogenic and mutualistic relationships with the host. Our hypothesis was that this strategy would identify genes that support one or the other lifestyle and furthermore would identify virulence genes not shared by beneficial members of the commensal microbiota, thereby increasing their potential applicability in vaccines.

Our detection of nonfunctional remnants of ancestral genes both in S. pneumoniae and in S. mitis strains supports the described evolutionary model and the concept that evolutionary changes on both sides were required to achieve their present relationship to the host. However, the demonstrated variation in the completeness of the elimination of certain genes suggests that adaptation is still an ongoing process (Table 1). Accordingly, ancestral genes encoding recognized virulence factors, like pneumolysin, autolysin, neuraminidases A and B (NanA and NanB), beta-galactosidase (*bgaA*), beta-*N*-acetylhexosaminidase (*strH*), and *endoD*, are still present in some S. mitis strains.

The extensive strain differences observed within all species (see Table S1 in the supplemental material) are in accordance with a core genome and accessory genes present only in a subset of clones (30, 69). This study expands on earlier studies (38, 40) by showing that even the cell wall teichoic acid structure (corresponding to the Lancefield group antigen) may vary between closely related members of the *Streptococcus* species examined (Table S3). Previous studies identified 13 regions of diversity in pneumococcal genomes, which account for greater than half the genomic diversity among isolates (30–32). Genes in some of these regions appear to be associated with virulence (32, 70), which may be part of the explanation of differences in the pathogenic potential of pneumococcal clones. Thus, the propensity of a clone to cause invasive disease is dependent on its serotype and its genomic content, both of which may undergo frequent changes (67). The 13 RDs were present in from one to nine of the 13 S. pneumoniae strains of this study, and seven of them were generally absent from genomes of the commensal species. Therefore, it is conceivable that the filters used in our comprehensive search for virulence-associated genes (present in at least 10 out of 13 S. pneumoniae strains and absent from at least 80% of S. mitis strains) resulted in the neglecting of some genes that may explain the differential pathogenic potentials of pneumococcal strains.

Our screen for genes associated with virulence identified 224 genes that fulfilled the filtering criteria (Table 1), of which 29 genes encoded proteins of unknown function. Many of the 224 genes form putative operons consisting of 2 to 16 genes in S. pneumoniae. We identified 101 loci, in which all component genes were either absent or present in the individual strains of commensal species. Among the 224 genes, 116 were absent from all S. mitis strains. Of these, 78 were also absent from S. oralis strains, and 49 genes were absent from all 40 strains of S. mitis, S. oralis, and *S. pseudopneumoniae* (Table 1). As shown in Table 1, the genes identified included recognized pneumococcal virulence factors such as pneumococcus surface protein A (PspA), the hyaluronidase locus, pneumolysin (Ply), autolysin (LytA), and choline-binding protein A (CbpA/PspC). All of these were also identified in one or more of the STM studies (10–12). It is interesting that CbpA was identified in STM studies (12) and is upregulated in experimental lung infections in mice (14). The protein binds factor H (FH), which minimizes the activity of the alternative pathway of complement and, therefore, the capacity of the isolate to evade opsonophagocytosis (71). However, CbpA binds to human FH but not to the FH proteins of mice (72), which suggests that the protein has other, unknown functions or mechanisms of action. The same is true for IgA1 protease, which was identified as a virulence factor in the STM experimental pneumonia study by Polissi et al. (10) despite the fact that it cleaves human IgA1 but not murine IgA (73).

Substantial direct and indirect evidence shows that expression of capsular polysaccharide is crucial to the pathogenic potential of S. pneumoniae. Therefore, it was surprising when we identified complete capsular polysaccharide biosynthesis operons and expressed capsules in virtually all strains of commensal viridans streptococci (16). Although some pneumococcal polysaccharide serotypes are far more commonly associated with disease, many of the polysaccharides expressed by commensal species are identical to recognized pneumococcal serotypes, including serotypes that are part of current vaccines (16, 74, 75). Within a given pneumococcal serotype, virulence appears to be related to the amount of capsular polysaccharide produced (76, 77). Like other bacterial pathogens, including Haemophilus influenzae serotype b (78), Neisseria meningitidis (79), and Klebsiella pneumoniae (80), S. pneumoniae has the capacity to up- and downregulate the production of capsular polysaccharide. One regulatory mechanism identified in S. pneumoniae is the invertible type I restriction-modiﬁcation system (*ivr* locus/SpnD39III), which previously was demonstrated to undergo rearrangements through sequence inversion in S. pneumoniae TIGR4 (33). Through genetic rearrangements and ensuing changes in specificity, the system regulates capsule expression by different patterns of DNA methylation (35). This system was missing from S. mitis and the other commensal species examined in this study (Table 1 and Table S1B). Thus, dynamic control of the expression of the capsule, which is essential for S. pneumoniae to thrive in niches in which amounts of capsule are differentially required (81), is missing from commensal species.

A second type 1 RM system showing direct-repeat *hsdS* loci (*tvr*) (SP_0886 to SP_0892) likewise is ubiquitous to S. pneumoniae (82). It also was present in *S. pseudopneumoniae* but not in any of the S. mitis strains (Table S1B). The function of this locus is still unknown (83), and the consequences of this difference remains to be identified.

In addition to their role as a defense strategy against the invasion of foreign DNA, emerging data indicate that RM systems serve a role in recombination, nutrition, and the generation of genetic diversity (84). Therefore, it is interesting that, apart from a few exceptional strains, S. mitis strains lacked the entire range of RM systems present in S. pneumoniae except for the type IV endonuclease (Table S1B).

Elimination of regulatory mechanisms appears to have been a major component of the adaptation to a mutualistic life style. As shown in Table 1 and Tables S1B to D, as many as 15 transcriptional regulators, two 2-component system (TCS) response regulators, and the mentioned RM systems are missing from the mutualistic species. Although many of these are part of lost operons, primarily carbohydrate transport systems, eight transcriptional regulators and SpnD39III were lost without loss of the apparent associated operons. Among these transcriptional regulators lacking in commensals are four involved in carbohydrate metabolism, including the galactose operon, tryptophan synthesis, phosphate transport, and unknown functions. Of particular interest is the absence from all commensal strains of the two 2-component system response regulators TCS04 (PnpR/S) and TCS06, which were shown to regulate transformation competence, fitness, immune evasion, and expression of PsaA (TCS04) and colonization, invasion, and CbpA expression (TCS06) (36). The importance of transcriptional regulators is in agreement with STM studies in mouse models of pneumonia and bacteremia (12). The exact function of the two pleiotropic regulators (PlcR) in S. pneumoniae is unknown. In Bacillus thuringiensis, a homologous PlcR regulates extracellular virulence gene expression (85). These results further support the concept that successful adaptation of S. mitis and other commensal streptococci to a harmonious relationship with the host relied on phenotypic stability rather than phenotypic flexibility.

The capacity to degrade, transport, and metabolize complex glycans has been identiﬁed as a key virulence mechanism in S. pneumoniae. These activities contribute to degradation of host polymers, including mucins, glycolipids, and hyaluronic acid in connective tissue, exposition and interaction with host cell receptors, and evasion of host immune defense (33, 34, 86). The pneumococcal genome encodes more than 40 known or predicted proteins that cleave glycosidic bonds in glycans, many of which are upregulated when pneumococci are in contact with lung epithelia (13). Although reliance on sugar transport and metabolism is a common feature of streptococci, it is striking that 14 glycosidases, at least eight glycosyltransferases, and 19 out of 32 carbohydrate uptake systems were missing in S. mitis and the other mutualistic species (Table 1 and Table S1E). The significantly reduced arsenal of these enzymes in the commensal species conceivably restricts the range of potential nutrients derived from host polymers and probably the range of niches that are accessible to the commensals, such as internal host tissues and circulation.

Recently, Trappetti et al. (87) demonstrated that autoinducer-2 (AI-2) signaling via FruA, a fructose-specific phosphoenolpyruvate-phosphotransferase system (PTS), enables S. pneumoniae to utilize galactose as a carbon source and upregulates the Leloir pathway, thereby leading to increased production of capsular polysaccharide and a hypervirulent phenotype. Although *fruA* and the Leloir pathway genes *galR*, *galK*, and *galT* are ubiquitous to all the genomes examined, *S. pseudopneumoniae* and S. mitis lack important genes involved in galactose uptake and metabolism, which may interfere with this regulatory mechanism. Galactose is the dominant carbon source available to pneumococci in the upper respiratory tract and is largely derived from host glycoconjugates by the sequential action of NanA (neuraminidase) and BgaA (β-galactosidase) (88, 89), both of which were absent from most strains of S. mitis.

In addition to the carbohydrate transporters, seven ABC transporters were absent from the mutualistic species (Table S1F). They included two inorganic phosphate (P_i_) transporters present in S. pneumoniae and *S. pseudopneumoniae* strains and an iron compound transporter system unique to S. pneumoniae (Table 1 and Table S1F). It is remarkable that the two phosphate uptake systems PstI (SP_2084-2088) and PstII (SP_1824-1830), their respective PhoU transcriptional regulators, and the associated two-component histidine transcriptional regulator PnpR/S (TCS04) are unique to S. pneumoniae and *S. pseudopneumoniae* (apart from four S. mitis strains having PstII). According to Zheng and coworkers (90), either the PstI or PstII P_i_ transporter is required in encapsulated pneumococci. Thus, a double Δ*pstI* Δ*pstII* mutation could not be created in encapsulated pneumococci. The regulated PstI and constitutive PstII P_i_ transport systems seem to act as a redundant failsafe to ensure that capsule biosynthesis is maintained during variations in P_i_ concentrations. A third cotransporter of sodium and phosphate (SP_0496; NptA) ensured normal growth of noncapsulated, Δ*pstI* Δ*pstII* mutant S. pneumoniae D39. It is conceivable that the latter low-affinity transporter, which is ubiquitous to all strains examined in this study, secures phosphate uptake in commensal streptococci, but it remains an enigma how this is compatible with the encapsulation of most commensal strains. It may result in, or require, less efficient capsular biosynthesis in commensal streptococci.

Strains of S. pneumoniae express from 13 to 16 choline-binding surface proteins that are linked to the cell wall by noncovalent interaction with phosphorylcholine residues in the wall teichoic acid. Four of these Cbps were unique or almost unique to pneumococci, i.e., pneumococcal surface protein A (PspA), CbpG, PcpA, and CbpA/PspC (Table 1). Their function as virulence factors is supported by an STM study of pneumonia in mice (12) and by the finding that they are significantly upregulated in the heart (15). Two of these, PspA and CbpA/PspC, were completely absent from the commensal species. PspA interferes with complement activation and binds lactoferrin, thereby protecting pneumococci from killing by components of the innate immune system. CbpA/PspC (also named SpsA and PbcA) is a multifunctional protein. It is the major adhesin of pneumococci (91, 92) and contributes to nasopharynx and lung colonization (91, 93), inhibits complement activation (94, 95), and promotes invasion by specific binding to the human polymeric immunoglobulin receptor (hpIgR) and its derivative secretory component (SC) of secreted IgA and IgM. It has been proposed that this interaction enables the bacteria to exploit the host cell polymeric immunoglobulin transcytosis machinery to promote their translocation across the mucosal barrier (92, 96). Moreover, PspC interacts directly with a laminin-specific integrin receptor ubiquitously expressed on vascular endothelial cells, contributing to invasive diseases, including pneumococcal meningitis (14). CbpG is also a multifunctional surface protein that, in the cell-attached or secreted forms, cleaves host extracellular matrix and in the cell-attached form participates in bacterial adherence (97). PcpA can mediate adherence of pneumococci to human nasopharyngeal and lung epithelial cells (98).

The general presence of the riboflavin biosynthesis operon in genomes of S. pneumoniae and *S. pseudopneumoniae* and absence from S. mitis and S. oralis is in agreement with a recent report (99). The importance of the operon in pneumococcal virulence was suggested by STM studies of experimental pneumonia in mice (11, 12). Previous studies found that riboflavin biosynthesis is essential for intracellular survival of Brucella abortus (100). Recent studies point to another role in pneumococci. Mucosa-associated invariant T (MAIT) cells represent an innate T-cell population abundant in the lung, blood, and liver, which recognize ligands generated by the microbial riboflavin synthesis pathway, presented via the major histocompatibility complex class I-related molecule (MR1) (96). It is unclear if this constitutes an advantage for the pneumococci, but absence from S. mitis and other commensal streptococci would be in agreement with their aim to retain a harmonious balance with the immune system.

Of the three iron ABC transporters described in the pneumococcal genome (33, 101–103), the Pit transporter located in a pathogenicity island together with phage-associated proteins (SP_1030-1040) was unique to S. pneumoniae (Table 1), while the remaining two were present in most commensal strains. There is functional overlap between the three, and studies by Brown et al. (96) showed that if the two iron transporters Piu and Pia are present, then Pit has only a relatively small role during growth *in vivo*. However, several studies of loss-of-function mutants in the Pit transporter, which is lacking in the commensal species, demonstrated impaired virulence in a mouse model of S. pneumoniae systemic infection and pneumonia (11, 12, 102).

Focus on S. mitis shows that, apart from eliminating the genes listed in Table 1, adaptation to a mutualistic relationship with the host apparently required preservation or acquisition of 25 genes lost or absent from S. pneumoniae. As noted also by Denapaite et al. (24), many of these genes were present as pseudogenes or in truncated form in S. pneumoniae, supporting their ancestral origin. Others presumably were acquired from other commensal oral streptococci. The 25 genes were present in 18 or more of the 20 S. mitis strains, often as multigene loci, and usually were present also in S. oralis and *S. infantis* but variably so in *S. pseudopneumoniae* (Table 2).

In accordance with their ecological preference, the mutualistic species possessed several genes that play a role in biofilm formation (Table 2). The *lrgA* and *lrgB* genes and their paralogs, *cidA* and *cidB*, were previously studied in Streptococcus mutans and Staphylococcus aureus. The Lrg operon has substantial impact on the ability of S. mutans to colonize and persist in dental biofilms and to survive under oxidative stress conditions (104, 105). Oxidative stress is one of the most important environmental variables affecting the environmental success of oral streptococci, as high oxygen concentrations disfavor their growth. Lack of the Lrg operon may be one reason why S. pneumoniae never colonizes dental biofilms and the oral cavity in front of the palatine arches (106). In S. aureus, the *lrg-cid* genes coordinate cell death and lysis during biofilm development, causing release of genomic DNA, which ultimately becomes a structural component of the biofilm matrix (107).

Two genes encoding proteins of the SarA family, with the characteristic Leu-Ala-Ala-Cys-Ser motif corresponding to the consensus cleavage site of precursors of bacterial lipopeptides (108), were unique to commensal species. The SarA gene (SK12261_0266) was flanked by an amino permease protein gene (SM12261_0268) also missing in pneumococcal genomes (Table 2). The *aliD* gene (SM12261_1517) is located immediately upstream of the *cps* locus in commensal species, in some strains together with the paralog *aliC.* The presence of pseudogenes of *aliC* and *aliD* in some pneumococcal strains (16, 109) supports their ancestral nature. The *aliD* and *aliC* genes are 42% identical (on a nucleotide level) to *aliA*, which is located immediately downstream of the *cps* locus in both pneumococci and the commensal streptococci and 50% identical to *sarA* in Streptococcus gordonii. Potential functional diversities of the SarA/AliA/AmiA proteins are not known, but SarA^−^ mutants in S. gordonii are defective in serum-induced aggregation, competence, growth on complex nitrogen sources, and ability to colonize the oral cavity (108).

It is remarkable that the three queuosine biosynthesis proteins QueC, QueD, and QueE, ubiquitous to S. mitis, *S. pseudopneumoniae*, and S. oralis, were absent from S. pneumoniae. Queuosine is a hypermodified guanosine analog, which, by posttranscriptional modification of the anticodon loop of tRNAs, is critical for efficient and accurate translation. The three genes have been identified in pneumococcal phage genomes (110, 111) but were absent from the 13 S. pneumoniae genomes examined in this study, apart from a truncated version of QueC consisting of the first 213 of 654 bp. It is not clear if and how pneumococci acquire queuosine and why lack of biosynthesis is associated with the virulent lifestyle.

The presence or absence of genes (Tables 1 and 2) supports the phylogenetic analysis (Fig. 1B) that *S. pseudopneumoniae* is an intermediary between S. pneumoniae and S. mitis, as previously suggested (112). Unfortunately, the clinical importance of *S. pseudopneumoniae* is not yet clear due to identification difficulties in traditional clinical microbiology laboratories (reference 1 and see below).

The comprehensive genome comparisons carried out in our study identified significant differences between strains, as reflected in the number of strain-specific CDS (107 to 273 among S. mitis) (Table S1A). There are two striking examples of such strain-specific differences. As noted before (24), the genome of S. mitis B6 is larger than most other S. mitis genomes (Table S1A) and includes an unusually large number of choline-binding proteins (*n* = 22), including a PdpA-related protein (2) and numerous mobile elements. Another example is S. oralis subsp. *tigurinus* AZ_3a, which harbored 369 strain-specific CDS (Table S1A). The strain carries several of the genes associated with virulence in S. pneumoniae, including the hyaluronidase operon, choline-binding proteins PcpA and CbpG, peptide ABC transporter, teichoic acid biosynthesis genes, and pilus 2 proteins (Tables S2, S4, and S5) (113). Strain AZ_3a was isolated from human blood (113). Subsequently, the taxon was isolated from a range of infections, but as supported by our data, the type endowed with multiple virulence factors is rare in the taxon (114). These unusual genomic patterns may be due to acquisition of genes by horizontal transfer or incomplete reductive evolution of the genomes. These examples, together with previous observations that strains of S. mitis may possess various numbers of recognized pneumococcal virulence factor genes and competence-regulated genes (17–20, 61), raise the question of whether such differences result in different virulence potentials, e.g., the propensity to cause endocarditis or bacteremia in leukemic patients. Our genome-based phylogenetic analysis and the presence of selected genes in an extended collection of S. mitis strains and relation to their clinical origin clearly demonstrated at least two major clusters and a number of apparently distinct lineages represented by only a few strains (Table S5). The close sequence similarity of the strikingly diverse *comM* allele and the locational similarity of the *zmpA* gene with S. pneumoniae suggest that cluster II of S. mitis is more closely related to S. pneumoniae than cluster I strains. Subclustering of the S. mitis population is in agreement with observations reported by Shelburne et al. (7), who studied 68 clinical strains using multilocus sequence analysis. According to Table S5, cluster I of our study was dominated by carrier isolates from the oral cavity and pharynx and from cases of endocarditis, while cluster II was dominated by isolates from cases of bacteremia.

S. mitis clones causing endocarditis or bacteremia in predisposed patients have an origin in the oral microbiota and gain passive access to the bloodstream through a compromised mucosal barrier due to local inflammation or to injury during dental procedures (115, 116). Thus, it is not surprising that carrier strains cluster with strains from infections. However, it is still possible that certain lineages of S. mitis are more prone to survive in the bloodstream and to cause infections, but neither capsule production nor IgA1 protease activity showed association with systemic disease. However, the findings in Table S5 suggest differences in clones that are capable of settling on the endocardium and clones causing a more general bacteremia in leukemic patients. A platelet-aggregating factor was reported to be implicated in the pathogenesis of endocarditis caused by some oral streptococci, in particular S. sanguinis, whereas S. mitis strains were found to be negative (117). Our data identified a platelet aggregation gene in nine out of ten isolates of S. mitis from cases of bacteremia, whereas only one out of 11 isolates from infective endocarditis had this gene (Table S5). A previous study identified platelet-aggregating activity mediated by phage-associated proteins (phage tail proteins) PblA and PblB in a strain of S. mitis isolated from endocarditis (55). In a study of nearly 500 pneumococcal genomes, Brueggemann and coworkers (63) identified *pblA* and *pblB* genes in 72% of prophages carried by pneumococci. Among our 13 strains, only Hungary19A-6 and P1031 possessed the two genes, and the genes were present in only three of the 40 strains of S. mitis.

Our study approach was based on the phylogenetic scenario resulting in the differentiation of an ancestral population of streptococci into two distinct life styles, i.e., the pathogenic S. pneumoniae and the mutualistic S. mitis. The strength of this approach is that the results are free from being affected by the potential problems of artificial animal infection models, which is particularly relevant with bacteria that show a high degree of adaptation to the human host. The initial detailed, manual comparison of genomes was based on a relatively limited number of genomes. However, with few exceptions, the subsequent screening of more than 7,500 genomes (Table S4) validated the results. Phylogenetic analysis of these genomes emphasized the importance of correct species assignment. Many of the genomes were mislabeled, a problem that was particularly severe for *S. pseudopneumoniae* (Fig. S5A). Supported by this comprehensive genome search, our approach confirmed a range of properties associated with virulence and added a number of properties not detected in previous studies, including several proteins of unknown function. The striking difference from commensal streptococci was the diversity and number of regulatory mechanisms, including regulation of capsule production, a significantly larger arsenal of enzymes involved in carbohydrate hydrolysis, and proteins known to interfere with innate immune factors. Their potential as vaccine components is increased by the fact that a direct impact on beneficial members of the commensal microbiota can be excluded. Furthermore, potential vaccine escape resulting from import by pneumococci of new alleles from closely related nonpathogenic streptococci is less likely.

## MATERIALS AND METHODS

### Genomes.

Genomes of 60 strains of S. pneumoniae, S. mitis, S. oralis (subsp. *oralis*, subsp*. tigurinus*, subsp. *dentisani*, and genomosubspecies 1), *S. infantis*, and one additional, closely related but singleton strain were included in the analyses (Table S1). The gap-free genomes of two of the S. mitis strains, including the designated type strain of the species, were generated as part of this study. The genome-sequenced strains were identified based on a phylogenetic analysis of core genome sequences (see below) and using the updated nomenclature presented by Jensen et al. (1).

The genome of S. mitis strain SK637 was sequenced by 454 pyrosequencing. Assembly resulted in seven contigs. This version of the genome was available in GenBank under accession number JPFX00000000. Gaps between the seven contigs were closed by targeted Sanger sequencing of PCR amplicons generated and sequenced with primers designed using the NCBI Primer-BLAST interface (https://www.ncbi.nlm.nih.gov/tools/primer-blast/) on the basis of regions flanking the gaps. The final gap-free genome sequence of SK637 is available in GenBank under accession number CP028415.

The genome of the type strain of S. mitis NCTC12261/SK142 was initially sequenced by Sanger shotgun sequencing and assembly, which resulted in 451 contigs. Subsequently, the genome was resequenced by 454 pyrosequencing, and assembly of the combination of Sanger and 454-generated data resulted in 24 contigs. This version of the genome was available at GenBank under accession number AEDX00000000. Most recently, we resequenced the SK142 genome using the Pacific Biosciences (PacBio) RS-II platform, and assembly of PacBio data alone resulted in a complete gap-free sequence of the genome. This gap-free sequence was aligned with the Sanger and 454 contigs in order to identify potential sequencing errors generated by one of the three technologies, and base calls supported by at least two of the three technologies were kept in the final genome sequence, which is available at GenBank under accession number CP028414.

### Annotation of genomes.

The SK142 and SK637 genomes were annotated using the automated CloVR Microbe pipeline (118). The annotation of a subset of genes of interest was manually checked and corrected where necessary.

### Comparison of genomes.

The genomes were examined and compared in a Sybil database constructed as described previously (28) and established as part of this study (accessible at http://sybil-clovr.igs.umaryland.edu/sybil/sybil_mitis_group_v2). To verify findings of genes/proteins that apparently were unique to particular taxa, all such protein sequences were tested for homologies in the nonredundant protein database at NCBI (https://blast.ncbi.nlm.nih.gov) using BLASTN or BLASTP.

### Phylogenetic analysis.

Phylogenetic analysis of core genome sequences of the 60 strains generated by the alignment produced in CLoVR was conducted in MEGA 7.0.18 (26) using the maximum parsimony and minimum evolution algorithms and in SplitsTree4 (version 4.14.4) (27). The validity of the trees generated by the analyses was tested by bootstrap analysis based on 500 replications. Cluster analyses of single genes or protein sequences were also conducted using the minimum evolution algorithm in MEGA 7.0.18.

Identification of zinc-metalloprotease species was achieved according to phylogenetic analysis based on amino acid sequences combined with the demonstrated *in vitro* cleaving activities as described previously (46).

To validate the initial findings on subclusters of S. mitis, we performed an alignment that included an additional 20 genomes of S. mitis that recently became publicly available. Whole-genome-level core SNP-based phylogenetic analysis of our 20 S. mitis genomes together with 20 publicly available S. mitis genomes, plus S. oralis Uo5 and S. pneumoniae TIGR4 and ATCC 700669 as outgroups, was performed. First, SNPs were identified within the core regions shared by all genomes using the In Silico Genotyper (ISG) pipeline (119) with default parameters. The ISG output of “clean unique variants,” a concatenated FASTA file of aligned core SNPs, was then used for phylogenetic analysis in SplitsTree (version 4.14.4) (21).

### Prophages.

The presence of prophage genes in the genomic sequences were detected using PHASTER (120).

### Phylogenetic validation of the identity of comprehensive genome collection and search of genes.

All available nucleotide sequence Multi-FASTA files of genome assemblies for strains labeled S. pneumoniae (number of unique strains, 7,398), *S. pseudopneumoniae* (*n* = 43), S. mitis (*n* = 89), S. oralis (*n* = 75), and *S. infantis* (*n* = 11) were downloaded from the NCBI RefSeq assembly archive on 10 May 2019 using ftp (ftp.ncbi.nlm.nih.gov/genomes/refseq/assembly_summary_refseq.txt). We used the database of >7,500 streptococcal genome assemblies to calculate a pairwise genome distance matrix using MASH v2.1 (https://www.ncbi.nlm.nih.gov/pubmed/27323842). The matrix was then converted to .meg format using an in-house script for loading into MEGA 7 (https://www.ncbi.nlm.nih.gov/pubmed/27004904), where a neighbor-joining tree was computed using pairwise deletion. The Newick output was then loaded into FigTree v1.4.3 http://tree.bio.ed.ac.uk/software/figtree/) to generate a midpoint-rooted circular tree.

The nucleotide sequences of the 224 genes that we identified as specific to S. pneumoniae, as well as those of the 25 genes specific to commensal streptococci, were searched against this large database of streptococcal genome assemblies. Searches were performed with BLASTN against genome sequences (not predicted gene sequences), and BLASTN hits were deemed representative of gene presence if alignments spanned at least 40% of the query sequence with at least 80% identity. While the short alignment length requirement of 40% was used to accommodate gaps and low-quality regions in draft genomes, the number of genes deemed present in genomes using these cutoffs represents a very conservative estimate of gene presence.

### Accession number(s).

The completed genomes were deposited in the NCBI database under the following accession numbers: Streptococcus mitis NCTC 12261, CP028414 (BioProject no. PRJNA173); Streptococcus mitis SK637, CP028415 (BioProject no. PRJNA242571).
