# Erratum for Kilian and Tettelin, “Identification of Virulence-Associated Properties by Comparative Genome Analysis of Streptococcus pneumoniae, *S. pseudopneumoniae*, S. mitis, Three S. oralis Subspecies, and *S. infantis*”

**DOI:** 10.1128/mBio.02520-19

**Published:** 2019-10-15

**Authors:** Mogens Kilian, Hervé Tettelin

**Affiliations:** a Department of Biomedicine, Aarhus University, Aarhus, Denmark; b Department of Microbiology and Immunology, Institute for Genome Sciences, University of Maryland School of Medicine, Baltimore, Maryland, USA

## ERRATUM

Volume 10, issue 5, e01985-19, 2019, https://doi.org/10.1128/mBio.01985-19. At initial publication, the wrong file was present for Fig. S3 in the supplemental material. The file was fixed online on 9 September 2019.

