# Supplementary material for: Identification of Virulence-Associated Properties by Comparative Genome Analysis of Streptococcus pneumoniae, S. pseudopneumoniae, S. mitis, Three S. oralis Subspecies, and S. infantis
Source: mBio. 2019 Sep 3;10(5):e01985-19. doi: 10.1128/mBio.01985-19 (PMC6722419; doi:10.1128/mBio.01985-19)
Supplement: Table S1 [file mBio.01985-19-st001.pdf]

Table S1A. Genomes of *Streptococcus* strains included in the study

Strain	Genome size (mb)	No. of contigs	No. of CDS	No. of unique CDS	Isolated from	Accession	Refer ence
S. pneumoniae TIGR4	2,16	1	2125	210	Human blood, Norway	AE005672	1
S. pneumoniae Hungary19A-6	2,25	1	2252	176	Hungary	CP000936	2
S. pneumoniae Taiwan19F-14	2,11	1	2110	141	Taiwan	CP000921	2
S. pneumoniae P1031	2,11	1	2135	141	Ghana	CP000920	2
S. pneumoniae 70585	2,18	1	2215	209	Bangladesh	CP000918	2
S. pneumoniae G54	2,08	1	2026	133	Clinical isolate, Italy	CP001015	3
S. pneumoniae ATCC 700669	2,22	1	2118	138	Spain	FM211187	4
S. pneumoniae JJA	2,12	1	2118	143	Brazil	CP000919	2
S. pneumoniae R6	2,04	1	2043	120	USA	AE007317	5
S. pneumoniae D39	2,05	1	1914	88	USA	CP000410	6
S. pneumoniae 670	2,24	1	2289	194	Spain	CP002176	2
S. pneumoniae CGSP14	2,21	1	2205	188	Pneumonia, Taiwan	CP001033	7
S. pneumoniae TCH8431/19A	2,09	1	2275	209	Respiratory tract, unknown	CP001993	8
S. mitis NCTC 12261/SK142T	1,87	1	1741	104	Human oral cavity	CP028414	9
S. mitis B6	2,15	1	2003	249	Unknown	NC_013853	10
S. mitis SK137_CCUG 35791	1,96	41	1963	164	Tooth surface	JPFS00000000	11
S. mitis SK271	1,94	14	1799	116	Tooth surface	JPGW00000000	11
S. mitis SK321_NCTC8034[1]	1,87	40	1799	119	Human throat	AEDT00000000	11
S. mitis SK564	2,03	35	1952	182	Human blood	AEDU00000000	11
S. mitis SK569_CCUG 62643	1,98	54	1850	125	Blood, leukemic child	AFUF00000000	13
S. mitis SK575_CCUG 62644	2,02	90	1937	158	Blood, leukemic child	AICU00000000	13
S. mitis SK578	2,08	16	1998	238	Blood, leukemic child	JPFY00000000	11
S. mitis SK579/CCUG 62641	1,98	82	1908	153	Blood, leukemic child	AJJL00000000	13
S. mitis SK597/CCUG 55094	2,03	109	1932	197	Human urethra	AEDV00000000	11
S. mitis SK608	2,13	15	1985	196	Healthy pharynx	JPFZ00000000	11
S. mitis SK616/CCUG 62642	1,99	126	1910	144	Human blood	AICR00000000	13
S. mitis SK629	2,21	27	2140	273	Human oral cavity	JPFU00000000	11
S. mitis SK637/CCUG 35816	1,94	1	1816	120	Human oral cavity	CP028415	9
S. mitis SK642	1,99	11	1879	198	Human oral cavity	JPFW00000000	11
S. mitis SK667	2,14	15	1985	161	Human blood	JPFV00000000	11
S. mitis SK1073/CCUG 47273[2]	2,11	54	2003	198	Human blood	AFQT00000000	13
S. mitis SK1080/CCUG 50731[3]	1,94	34	1854	191	Blood, leukemic child	AFQV00000000	13
S. mitis SK1126	1,93	9	1805	107	Human oral cavity	JPFT00000000	11
S. oralis subsp. *oralis* ATCC 35037T	1,88	25	1750	105	Human oral cavity	AEDW00000000	11
S. oralis subsp. *oralis* Uo5	1,96	1	1839	124	Unknown	NC_015291.1	12
S. oralis subsp. oralis SK10/NCTC7864	1,94	39	1879	130	Human blood	AJKO00000000	13
S. oralis subsp. oralis SK141	1,87	11	1846	135	Human oral cavity	JPGA00000000	11
S. oralis subsp. *oralis* SK143	2,04	7	1960	170	Human oral cavity	JPGB00000000	11
S. oralis subsp. *oralis* SK610	1,99	31	1895	136	Healthy human pharynx	AJKQ00000000	13

S. oralis subsp. *dentisani* 7747T	1,87	6	1798	106	Human tooth,caries free surface	CAUK00000000	14
S. oralis subsp. *dentisani* 7746	1,97	8	1921	149	Human tooth, caries free surface	CAUJ00000000	14
S. oralis subsp. *dentisani* F0392	1,92	2	1861	154	Human specimen	AFUO00000000	13
S. oralis subsp. *dentisani* SK95	2,02	66	2006	211	Human oral cavity	AFUB00000000	13
S. oralis subsp. *dentisani* F0407	1,85	58	1876	203	Human oral cavity	AGAE00000000	13
S. oralis subsp. *tigurinus* AZ_3aT	2,18	22	2114	369	Human blood	AORU00000000	15
S. oralis subsp. *tigurinus* SK255[4]	2,01	63	2004	148	Human oral cavity	AFNM00000000	13
S. oralis subsp. *tigurinus* SK304/H1 3	2,02	25	2007	199	Human oral cavity	ALJN00000000	13
S. oralis subsp. *tigurinus* SK313/ATCC 15914 4	1,89	5	2022	133	Healthy pharynx	AFUU01000001	13
S. oralis subsp. *tigurinus* SK1074[5]	1,85	20	1763	127	Human blood	AICT00000000	13
S. oralis genomosp. 1 ATCC 6249[6]	1,9	20	1793	132	Human blood, SBE	AEEN00000000	8
S. infantis ATCC700779T	1,86	53	1876	246	Human oral cavity	AJTA00000000	13
S. infantis SK140/CCUG 62652	1,9	41	1856	239	Human oral cavity	AJML00000000	13
S. infantis SK970	1,95	9	2144	257	Human blood	AFUT00000000	13
S. infantis SK1076/CCUG 42984	1,72	30	1599	125	Human blood	AFNN00000000	13
*S. infantis*SPAR10 5	1,76	10	1621	112	Human blood	ALCH00000000	16
S. infantis SK1302	1,79	148	2111	242	Unknown	AEDY00000000	11
S. pseudopneumoniae ATCC BAA-960	2,09	253	2113	156	Human clinical specimen	AICS00000000	13
S. pseudopneumoniae IS7493	2,19	1	2159	204	Human sputum	CP002925	17
S. pseudopneumoniae SK674/ CCUG 62647	2,08	109	2043	181	Healthy pharynx	AJKE00000000	13
Streptococcus species SK643/CCUG 62653	1,86	31	1788	188	Human oral cavity	AJMM00000000	13

[1]Listed as *Lactococcus lactis* in NCTC database

[2]Listed as *Streptococcus oralis* in CCUG database

[3]Listed as *Streptococcus pneumoniae* in CCUG database

[4]Genome sequence deposited as *Streptococcus oralis*

[5]Genome sequence deposited as *Streptococcus mitis*

[6]Strain listed as *Streptococcus oralis* in the ATCC catalogue and genome sequence deposited as *Streptococcus mitis*

REFERENCES

1. **Tettelin H, Nelson KE, Paulsen IT, Eisen JA, Read TD, Peterson S, Heidelberg J, DeBoy RT, Haft DH, Dodson RJ, Durkin AS, Gwinn M, Kolonay JF, Nelson WC, Peterson JD, Umayam LA, White O, Salzberg SL, Lewis,MR, Radune D, Holtzapple E, Khouri H, Wolf AM, Utterback TR, Hansen CL, McDonald LA, Feldblyum TV, Angiuoli S, Dickinson T, Hickey EK, Holt IE, Loftus BJ, Yang F, Smith HO, Venter JC, Dougherty BA, Morrison DA, Hollingshead SK, Fraser CM.** 2001. Complete genome sequence of a virulent isolate of *Streptococcus pneumoniae*. Science **293:** 498-506.
2. **Donati C, Hiller NL, Tettelin H, Muzzi A, Croucher NJ, Angiuoli SV, Oggioni M, Dunning Hotopp JC, Hu FZ, Riley DR, Covacci A, Mitchell TJ, Bentley SD, Kilian M, Ehrlich GD, Rappuoli R, Moxon ER, Masignani V.** 2010. Structure and dynamics of the pan-genome of *Streptococcus pneumoniae* and closely related species. Genome Biol; **11(10):**R107. doi: 10.1186/gb-2010-11-10-r107.

3. **Dopazo J, Mendoza A, Herrero J, Caldara F, Humbert Y, Friedli L, Guerrier M, Grand-Schenk E, Gandin C, de Francesco M, Polissi A, Buell G, Feger G, García E, Peitsch M, García-Bustos JF.** 2001. Annotated draft genomic sequence from a *Streptococcus pneumoniae* type 19F clinical isolate. Microb Drug Resist; **7:** 99-125.
4. **Croucher NJ, Walker D, Romero P, Lennard N, Paterson GK, Bason NC, Mitchell AM, Quail MA, Andrew PW, Parkhill J, Bentley SD, Mitchell TJ.** 2009. Role of conjugative elements in the evolution of the multidrug-resistant pandemic clone *Streptococcus pneumoniae* Spain23F ST81. J Bacteriol; **191:**1480-1489. doi: 10.1128/JB.01343-08.
5. **Hoskins JA, Alborn W Jr, Arnold J, Blaszczak L, Burgett S, DeHoff BS, Estrem S, Fritz L, Fu D-J, Fuller,W, Geringer C, Gilmour R, Glass JS, Khoja H, Kraft A, LaGace R, LeBlanc DJ, Lee LN, Lefkowitz EJ, Lu J, Matsushima P, McAhren S, McHenney M, McLeaster K, Mundy C, Nicas TI, Norris FH, O'Gara M, Peery R, Robertson GT, Rockey P, Sun P-M, Winkler ME, Yang Y, Young-Bellido M, Zhao G, Zook C, Baltz RH, Jaskunas SR, Rosteck PR Jr, Skatrud PL, Glass JI.** 2001. Genome of the bacterium *Streptococcus pneumoniae* strain R6 J Bacteriol **183:** 5709-5717.
6. **Lanie JA, Ng WL, Kazmierczak KM, Andrzejewski TM, Davidsen TM, Wayne KJ, Tettelin H, Glass JI, Winkler ME.** 2007. Genome sequence of Avery's virulent serotype 2 strain D39 of *Streptococcus pneumoniae* and comparison with that of unencapsulated laboratory strain R6. J Bacteriol; **189:** 38-51.
7. **Ding F, Tang P, Hsu MH, Cui P, Hu S. Yu J, Chiu CH.** 2009. Genome evolution driven by host adaptations results in a more virulent and antimicrobial-resistant Streptococcus pneumoniae serotype 14. BMC Genomics **10,** 158.
8. **Muzny DM, Qin X, Buhay CJ, Dugan-Rocha S, Ding Y, Chen G, Hawes AC, Holder M, Jhangiani SN, Johnson AJ, Khan ZM, Li Z, Liu W, Liu X, Perez LM, Shen H, Wang Q, Watt JE, Xi L, Xin Y, Zhou J, Deng J, Jiang H, Liu Y, Qu J, Song X-Z, Zhang L, Villasana D, Liu J, Liyanage D, Lorensuhewa LM, Robinson T, Song A, Song B-B, Dinh HH, Thornton R, Coyle MD, Francisco L, Jackson L, Javaid M, Korchina V, Kovar CL, Mata R, Mathew T, Ngo R, Nguyen L, Nguyen N, Okwuonu G, Ongeri F, Pham C, Simmons D, Wilczek-Boney KB, Hale W, Jakkamsetti A, Opheim D, Pham P, Lucas FS , Warren J, Zhang J, Zhao Z, Zhou C, Zhu D, Lee SL, Bess CM, Blankenburg KP, Forbes L, Fu Q, Gubbala S, Hirani K, Jayaseelan JC, Lara F, Munidasa M, Palculict T, Patil SS, Pu L-L, Saada N, Tang L-Y, Weissenberger GM, Zhu Y, Hemphill L, Shang Y, Youmans B, Ayvaz T, Ross M, Santibanez J, Aqrawi P, Gross S, Joshi V, Fowler G, Nazareth L, Reid J, Worley KC, Petrosino J, Highlander S, Gibbs,RA.** 2010. Direct Submission. Human Genome Sequencing Center, Baylor College of Medicine, One Baylor Plaza, Houston, TX 77030, USA.
9. **Kilian M, Tettelin H.** This study.
10. **Denapaite,D., Brückner,R., Nuhn,M., Reichmann,P., Henrich,B., Maurer,P., Schaehle,Y., Selbmann,P., Zimmermann,W., Wambutt,R., Hakenbeck,R.** 2010. The genome of Streptococcus mitis B6 - what is a commensal? PLoS ONE **5(2)**: e9426.
11. **Kilian M, Poulsen K, Blomqvist T, Håvarstein LS, Jørgensen M, Tettelin H, Sørensen UBS.** 2008. Evolution of *S. pneumoniae* and its close commensal relatives. PLoS ONE; **3(7):** e2683.
12. **Reichmann P, Nuhn M, Denapaite D, Bruckner R, Henrich B, Maurer P, Rieger M, Klages S, Reinhard R, Hakenbeck R.** 2011. Genome of *Streptococcus oralis* strain Uo5. J Bacteriol **193:** 2888-2889.
13. **Durkin AS, McCorrison J, Torralba M, Gillis M, Methe B, Sutton G, Nelson KE.** 2012. Direct Submission. The J. Craig Venter Institute, 9704 Medical Center Dr., Rockville, MD 20850, USA.
14. **Camelo-Castillo A, Benitez-Paez A, Belda-Ferre P, Cabrera-Rubio R, Mira A.** 2014. *Streptococcus dentisani* sp. nov., a novel member of the mitis group. Int J Syst Evol Microbiol 64: 60-65.
15. **Gizard Y, Zbinden A, Schrenzel J, Francois P.** 2013. Whole-genome sequences of *Streptococcus tigurinus* type strain AZ_3a and *S. tigurinus* 1366, a strain causing prosthetic joint infection. Genome Announc **1(2)**: E00210-12.
16. **Chancey S, Kumar N, Sengamalay N, Matthews C, Hine E, Pallavajjal A, Abolude O, Daugherty SC , Parankush, SP, Sadzewicz L, Tallon LJ, Farley MM, Baughman W, McGee L, Stephens DS, Tettelin H.** 2012. Direct Submission. Institute for Genome Sciences, The University of Maryland School of Medicine, 801 W Baltimore St, Baltimore, MD 21201, USA.
17. **Shahinas D, Tamber GS, Arya G, Wong A, Lau R, Jamieson F, Ma JH, Alexander DC, Low DE, Pillai DR**. 2011. Whole-genome sequence of *Streptococcus pseudopneumoniae* isolate IS7493. J Bacteriol **193:** 6102-6103.

Table S1B. DNA restriction modification systems in the examined *Streptococcus* genomes.

	SP (locus number)																			
	Type I										Type II								Type IV	
Strain	504	505	507	508	509	510	886	887	891	892	1221	1222	1430	1431	1849	1850	1935	1936	1250	1251
S. pneumoniae TIGR4	+	+	+	+	+	+	+	+	+	+	+	+	+	+	+	+	+	+	+	+
S. pneumoniae Hungary19A	+	+	+	+	+	+	+	+	+	+	-	-	+	+	-	-	+	+	+	+
S. pneumoniae Taiwan19F	+	+	+	+	+	+	+	+	+	+	-	-	+	+	+	+	+	+	+	+
S. pneumoniae P1031	+	+	+	+	+	+	+	+	+	+	-	-	+	+	-	-	+	+	+	+
S. pneumoniae 70585	+	+	+	+	+	+	+	+	+	+	-	-	+	+	-	-	+	+	+	+
S. pneumoniae G54	+	+	+	+	+	+	+	+	+	+	-	-	+	+	-	-	+	+	+	+
S. pneumoniae ATCC 700669	+	+	+	+	+	+	+	+	+	+	-	-	+	+	-	-	+	+	+	+
S. pneumoniae JJA	+	+	+	+	+	+	+	+	+	+	-	-	+	+	+	+	+	+	+	+
S. pneumoniae R6	+	+	+	+	+	+	+	+	-	+	+	+	+	+	+	+	+	+	+	+
S. pneumoniae D39	+	+	+	+	+	+	+	+	-	+	+	+	+	+	+	+	+	+	+	+
S. pneumoniae 670	+	+	+	+	+	+	+	+	+	+	-	-	+	+	-	-	+	+	+	+
S. pneumoniae CGSP14	+	+	+	+	+	+	+	+	+	+	-	+	+	+	+	+	+	+	+	+
S. pneumoniae TCH8431/19A	+	+	+	+	+	+	+	+	+	+	-	-	-	-	+	+	+	+	+	+
S. pseudopneumoniae ATCCBAA-960	-	+	+	+	+	+	+	+	+	+	+	+	+	+	+	+	+	+	-	-
S. pseudopneumoniae IS7493	-	-	+	+	+	+	+	+	+	+	+	+	+	+	-	-	-	-	+	+
S. pseudopneumoniae SK674	-	+	+	+	+	+	+	+	+	+	+	+	+	+	-	-	+	+	-	-
S. mitis NCTC 12261^T	-	-	-	-	-	-	-	-	-	-	-	-		-	-	-	-	-	-	-
S. mitis B6	-	-	-	-	-	-	-	-	-	-	-	-	-	-	-	-	-	-	-	-
S. mitis SK137	-	-	-	-	-	-	-	-	-	-	-	-	-	-	-	-	-	-	-	-
S. mitis SK271	-	-	-	-	-	-	-	-	-	-	-	-	-	-	-	-	-	-	-	-
S. mitis SK321	-	-	-	-	-	-	-	-	-	-	-	-	-	-	+	+	-	-	+	+
S. mitis SK564	-	-	-	-	-	-	-	-	-	-	-	-	-	-	-	-	+	+	+	+
S. mitis SK569	-	-	-	-	-	-	-	-	-	-	-	-	-	-	-	-	-	-	-	-
S. mitis SK575	-	-	-	-	-	-	-	-	-	-	-	-	-	-	-	-	-	-	-	-
S. mitis SK578	-	-	-	-	-	-	-	-	-	-	-	-	-	-	-	-	-	-	+	+

S. mitis SK579	-	-	-	-	-	-	-	-	-	-	-	-	-	-	-	-	-	-	-	-
S. mitis SK597	-	-	-	-	-	-	-	-	-	-	-	-	-	-	-	-	-	-	-	-
S. mitis SK608	-	-	-	-	-	-	-	-	-	-	-	-	-	-	-	-	-	-	+	+
S. mitis SK616	-	-	-	-	-	-	-	-	-	-	-	-	-	-	-	-	-	-	-	-
S. mitis SK629	-	-	-	-	-	-	-	-	-	-	-	-	-	-	+	+	-	-	-	-
S. mitis SK637	-	-	-	-	-	-	-	-	-	-	-	-	-	-	-	-	-	-	+	+
S. mitis SK642	-	-	-	-	-	-	-	-	-	-	-	-	-	-	-	-	-	-	-	-
S. mitis SK667	-	-	-	-	-	-	-	-	-	-	-	-	-	-	-	-	-	-	-	-
S. mitis SK1073	-	-	-	-	-	-	-	-	-	-	-	-	-	-	-	-	-	-	-	-
S. mitis SK1080	-	-	-	-	-	-	-	-	-	-	-	-	-	-	-	-	-	-	-	-
S. mitis SK1126	-	-	-	-	-	-	-	-	-	-	-	-	-	-	-	-	-	--	-	-
S. oralis ATCC 35037^T	-	-	-	-	-	-	-	-	-	-	+	+	+	-	-	-	-	-	+	+
S. oralis Uo5	-	-	-	-	-	-	+	+	+	+	-	-	-	-	-	-	-	-	+	+
S. oralis SK10	-	-	-	-	-	-	-	-	-	-	-	-	-	-	-	-	-	-	+	+
S. oralis SK141	-	-	-	-	-	-	-	-	-	-	-	-	-	-	-	-	-	-	+	+
S. oralis SK143	-	-	-	-	-	-	-	-	-	-	-	-	-	-	-	-	-	-	-	-
S. oralis SK610	-	-	-	-	-	-	-	-	-	-	-	-	-	-	-	-	-	-	+	+
S. oralis subsp. *tigurinus* AZ_3a^T	-	-	-	-	-	-	-	-	-	-	-	-	-	-	-	-	-	-	+	+
S. oralis subsp. *tigurinus* SK255	-	-	-	-	-	-	-	-	-	-	-	-	-	-	+	+	-	-	+	+
S. oralis subsp. *tigurinus* SK304	-	-	-	-	-	-	-	-	-	-	-	-	-	-	-	-	-	-	+	+
S. oralis subsp. *tigurinus* SK313	-	-	-	-	-	-	+	+	+	+	-	-	-	-	-	-	-	-	+	+
S. oralis subsp. *tigurinus* SK1074	-	-	-	-	-	-	+	-	+	+	-	-	-	-	-	-	-	-	+	+
S. oralis subsp. *dentisani* 7747^T	-	-	-	-	-	-	+	+	+	+			-	-	-	-	-	-	-	-
S. oralis subsp. dentisani 7746	-	-	-	-	-	-	+	+	+	+			-	-	-	-	-	-	+	+
S. oralis subsp. dentisani SK95	-	-	-	-	-	-	+	+	+	+			-	-	-	-	-	-	+	+
S. oralis subsp. dentisani F0392	-	-	-	-	-	-	(+)	-	-	-			-	-	-	-	-	-	-	-
S. oralis subsp. dentisani F0407	-	-	-	-	-	-	-	-	-	-			-	-	-	-	-	-	-	-
S. oralis genomosubsp. ATCC 6249	-	-	-	-	-	-	-	-	-	-	-	-	-	-	-	-	-	-	+	+
S. infantis ATCC 700779	+	-	+	+	+	+	-	-	-	-	-	-	-	-	-	-	-	-	+	+
S. infantis SK970	-	-	-	-	-	-	-	-	-	-	-	-	-	-	-	-	-	-	+	+
S. infantis SK1076	-	-	-	-	-	-	-	-	-	-	-	-	-	-	-	-	-	-	-	-
S. infantis SPAR10[5]	-	-	-	-	-	-	-	-	-	-	-	-	-	-	-	-	-	-	+	+

S. infantis SK1302	-	-	-	-	-	-	+	+	+	+	-	-	-	-	-	-	-	-	(+)	+
S. infantis SK140	-	-	-	-	-	-	-	-	-	-	-	-	-	-	-	-	-	-	-	-
Streptococcus species SK643	-	-	-	-	-	-	-	-	-	-	-	-	-	-	-	-	-	-	-	-

Table S1C. Two-component system (TCS) response regulators in *S. pneumoniae* and related species

Reference TIGR4	Name	*S. pneumoniae* N = 13	*S. pseudo-pneumoniae* N = 3	*S. mitis* N = 20	*S. oralis* subsp.			*S. infantis* N = 6	Regulates
					oralis N =6	*tigurinus* N = 5	*dentisani* N = 5		
SP_0083-4	SaeR/S TCS08	13/13	3/3	20/20	0/6	0/5	0/5	2/6	Pathogenesis and fitness; pilus expression (1, 2)
SP_0155-6	YesN/M TCS07	12/13	3/3	15/20	6/6	5/5	5/5	6/6	Fitness (1, 2)
SP_0376 (orphan)	ArlR TCS14	13/13	3/3	20/20	6/6	5/5	5/5	6/6	Virulence (1, 2)
SP_0386-87	LiaS/R TCS03	13/13	3/3	20/20	6/6	5/5	5/5	6/6	Antibiotic resistance and stress protection (1, 2)
SP_0526-27	BlpR/H TCS13	13/13	3/3	20/20	0/6	1/5	1/5	1/6	Virulence (2)
SP_0603-4	VncR/S TCS10	13/13	3/3	20/20	6/6	5/5	5/5	6/6	Antibiotic resistance (2)
SP_0661-2	ZmpR/S TCS09	13/13	3/3	20/20	6/6	5/5	5/5	0/6	Virulence, Zmp expression, thereby surface exposure of surface proteins including choline-binding proteins (3, 4)
SP_0798-9	CiaR/H TCS05	13/13	3/3	20/20	6/6	5/5	5/5	6/6	Regulated HtrA expression; important for systemic virulence in mice, infective endocarditis (5-7)
SP_1226-7	VicR/K TCS02	13/13	3/3	20/20	6/6	5/5	5/5	6/6	Essential; Redox/energy sensing; cell wall integrity; fatty acid biosynthesis; Expression of PspA (2)
SP_1632-3	TCS01	13/13	3/3	20/20	6/6	5/5	5/5	3/6	Virulence (2)
SP_2000-1	TCS11	13/13	3/3	19/20	6/6	5/5	5/5	6/6	Biofilm formation (2)
SP_2082-3	PnpR/S TCS04	13/13	0/3	0/20	0/6	0/5	0/5	0/6	Genetic competence, fitness, immune evasion; expression of PsaA (2)
SP_2192-3	TCS06	13/13	0/3	0/20	0/6	5/5	0/5	0/6	Colonization and invasion, CbpA (SP_2190) expression (8). CbpA not in *S. oralis* subsp. *tigurinus*
SP_2235/6	ComE/D TCS12	13/13	3/3	20/20	6/6	5/5	5/5	6/6	Genetic competence (2)

REFERENCES

1. **Throup JP, Koretke KK, Bryant AP, Ingraham KA, Chalker AF, Ge Y, Marra A, Wallis NG, Brown JR, Holmes DJ, Rosenberg M, Burnham MK.** 2000. A genomic analysis of two-component signal transduction in *Streptococcus pneumoniae*. Mol Microbiol **35:**566-576.
2. **Paterson GK, Blue CE, Mitchell TJ.** 2006. Role of two-component systems in the virulence of *Streptococcus pneumoniae*. J Med Microbiol; **55:** 355-363.
3. **Hendriksen WT, Silva N, Bootsma HJ, Blue CE, Paterson GK, Kerr AR, de Jong A, Kuipers OP, Hermans PW, Mitchell TJ.** 2007. Regulation of gene expression in *Streptococcus pneumoniae* by response regulator 09 is strain dependent. J Bacteriol; **189:** 1382-1389.
4. **Novak R, Cauwels A, Charpentier E, Tuomanen E.** 1999. Identification of a *Streptococcus pneumoniae* gene locus encoding proteins of an ABC phosphate transporter and a two-component regulatory system. J Bacteriol; **181:** 1126-1133.
5. **Mascher T, Zahner D, Merai M, Balmelle N, de Saizieu AB, Hakenbeck R.** 2003. The *Streptococcus pneumoniae cia* regulon: CiaR target sites and transcription profile analysis. J Bacteriol **185:** 60-70.
6. **Ibrahim YM, Kerr AR, McCluskey J, Mitchell TJ.** 2004. Control of virulence by the two-component system CiaR/H is mediated via HtrA, a major virulence factor of *Streptococcus pneumoniae*. J Bacteriol; **186:** 5258-5266.
7. **Trihn M, Ge X, Dobson A, Kitten T, Munro CL, Xu P.** 2013. Two-component system response regulators involved in virulence of *Streptococcus pneumoniae* TIGR4 in infective endocarditis. PLoS ONE; **8,1:**254320.
8. **Standish AJ, Stroeher UH, Paton JC.** 2005. The two-component signal transduction system RR06/HK06 regulates expression of *cbpA* in *Streptococcus pneumoniae*. Proc Natl Acad Sci USA **102:** 7701-7706.

Table S1D. Transcriptional regulator genes in *S. pneumoniae* TIGR4 and their presence in other strains of the study.

TIGR4 locus	Type and genomic context	Presense in *S. pneumoniae*	Presense in other strains
SP_0191	Transcriptional regulator, Spx	13/13	All strains except *S. infantis*
SP_0246/7	Transcriptional regulator, DeoR family, putative sorbitol operon regulator, part of PTS system	13/13	*S. mitis* SK321, SK597, SK1073, SK1080
SP_0306	Transcriptional regulator; hyaluronidase, cellobiose metabolism putative , smrD	8/13	*S. pseudopneumoniae* (3/3)
SP_0330	Sugar-binding transcriptional regulator RegR, repression of glucuronidase expression	13/13	*S. oralis* AZ_3a, SK313, SK1074
SP_0473	Transcriptional regulator (virulence), MgrA, PTS system, cellobiose-specific IIC component	10/13	*S. mitis* SK1080
SP_0716	Transcriptional regulator, TENA/THI-4 family protein, associated with ABC transporter	13/13	*S. pseudopneumoniae* (3/3), *S. mitis* (16/20), *S. oralis* subsp. *oralis* (3/5)
SP_0789	Transcriptional regulator, PadR family, associated with oxidoreductase	13/13	*S. pseudopneumoniae* (3/3), *S. mitis* (15/20)
SP_0927	Transcriptional regulator, LysR family, lipoprotein signal peptidase	13/13	All strains
SP_1057	Transcriptional regulator PlcR, ABC transporter	1/13	*S. mitis* (6/20)
SP_1227	Transcriptional regulator, YycF, ion transport	13/13	All strains
SP_1433	Transcriptional regulator, AraC family, part of ABC transporter system	11/13	None
SP_1774	Transcriptional regulator, MarR family, thioredoxin OBS!	13/13	All strains, queuosine biosynthesis in *S. mitis, S. pseudopneumoniae, S. oralis* and *S. infantis*
SP_1809	Transcriptional regulator, associated with tryptophan synthesis SP_1810-17	11/13	*S. pseudopneumoniae* (3/3), *S. mitis* SK1080
SP_1821	Sugar-bindingtranscriptional regulator, LacI family, associated with ABC transporter	13/13	*S. pseudopneumoniae* (3/3), *S. mitis* SK575, SK597, SK667, SK1073, *S. oralis* F-0392, SK304, *S. infantis* SK1302
SP_1856	Transcriptional regulator, MerR family, galactose operon	13/13	*S. pseudopneumoniae* (3/3), *S. mitis* NCTC12261, SK271, SK1073, *S. oralis* ATCC35037, SK141, Az_3a, SK1074
SP_1899	Msm (Multiple sugar metabolism) operon regulatory protein, AraC family, associated with alpha-galactosidase?	13/13	*S. mitis* NCTC12261, SK597, SK608, *S.oralis* 65%
SP_1920	Transcriptional regulator, MarR family, associated with ABC transporter (pseudogene in *S. pneumoniae*)	(13/13)	All strains
SP_1936	Putative Type II restriction-modification system regulatory protein	12/13	*S. pseudopneumoniae* ATCC_BAA_960, SK674, *S. mitis SK*564
SP_1942	Transcriptional regulator; Regulator of lytRABC operon; LCP family protein; proposed role: transferring of TA subunits to the peptidylglycan or to diacyl-glycerol; biosynthesis of teichoic acids; biofilm regulatory protein A	13/13	*S. pseudopneumoniae* (3/3), all *S. mitis* (20/20), all *S. infantis* (5/5), SK140, SK643

SP_1946	Transcriptional regulator PlcR (pleiotropic transcriptional regulator); unknown association	12/13	*S. mitis* SK12261; SK629
SP_1989	Transcriptional regulator PlcR	13/13	*S. mitis* SK608, SK642
SP_1999	Transcriptional regulator CcpA; pleiotropic, optimizes basic metabolic processes	13/13	All strains
SP_2020	Transcriptional regulator, GntR family, associated with PTS system	13/13	None
SP_2088/90	Phosphate transport system regulatory protein PhoU, PTS system, interrupted by transposase in TIGR4	13/13	*S. pseudopneumoniae* (3/3); *S. mitis* SK575, SK667, SK1073
SP_2172	Transcriptional regulator, sugar specific, ABC transporter	13/13	All strains

Table S1E. Carbohydrate uptake systems in *S. pneumoniae* and their presence in related species.

Locus TIGR4	Family	Name	Substrates	*S. pneumoniae*	*S. pseudopneumoniae*	*S. mitis*	*S. oralis*	*S. infantis*
SP_0061-4	PTS-Man		galactose	+	+	+	+	+
SP_0090-1	ABC CUT1			+	+	+	+	+
SP_0135-9	ABC-CUT1		GlcNAc	+	-	-	-	-
SP_0248-50	PTS-Lac			+	-	4/20	-	-
SP_0282-4	PTS-Man	*manLMN*	glucose, mannose, fructose, GlcNAc	+	+	+	+	+
SP_0305-8-10	PTS-Lac	*celBCD*	β-glucosides	7/13	1/3	-	-	-
SP_0321-5	PTS-Man		sulfated glycosamino-glycans	+	-	-	3/17	-
SP_0394-6	PTS-Fru	*mtlAF*	mannitol	10/13	+	-	-	-
SP_0474-6-8	PTS-Lac			10/13	1/3	-	-	-
SP_0577	PTS-Glc	*bglP*	β-glucosides	12/13	+	1/20	-	-
SP_0645-7	PTS-Gat	*gatABC*	galactose, lactose, galactiol	+	+	+	+	+
SP_0758	PTS-Glc	*malT*	maltose	+	+	+	+	+
SP_0845-8	ABC CUT2		ribonucleoside	+	+	+	+	+
SP_0877	PTS-Fru	*fruA*	fructose	+	+	+	+	+
SP_1185-6	PTS-Lac	*lacEF-2*	lactose, tagatose	+	+	+	+	+
SP_1197-8	PTS-Fru		fructose	+	-	-	-	-
SP_1328	Symporter	*ntp/app*	aminosugars	7/13	-	1/20	-	-
SP_1491	Facilitator		glycerol	+	+	+	+	+
SP_1617-9	PTS-Fru		pentoses	7/13	-	-	-	-
SP_1681-3	ABC CUT1		NeuNAc	+	+	+	+	+
SP_1684	PTS-Glc			+	+	1/20	-	-
SP_1688-90	ABC CUT1		NeuNAc	7/13	+	8/20	-	-
SP_1722	PTS_Glc	*scrH*	sucrose	+	+	+	+	+
SP_1796-8	ABC CUT1	*susXT1T2*	sucrose	7/13	+	-	-	-
SP_1884	PTS Glc		threhalose	+	+	2/20	6/17	-
SP_1895-7	ABC CUT1	*rafGFE*	α-galactosides	+	-	-	-	-
SP_2022-4	PTS-Lac		β-glucosides	+	-	-	-	-
SP_2036-8	PTS-Asc		ascorbate	+	-	7/20	1/17	-
SP_2108-10	ABC CUT1	*malXCD*	oligosaccharides	+	+	+	+	+
SP_2129-30	PTS-Asc		pentoses	+	+	7/20	-	-
SP_2161-4	PTS-Man		fucose, L-arabinose	9/13	-	2/20	-	-
SP_2184	Facilitator	*glpF*	glycerol	+	+	+	8/17	-

Table S1F. ABC transporters other than carbohydrate transporters specific to *S. pneumoniae*

Locus TIGR4	Proteins, putative function	*S. pneumoniae*	*S. pseudo-pneumoniae*	*S. mitis*	*S. oralis*	*S. infantis*
SP_0703-7	Peptide ABC transporter system: lysyl-tRNA synthetase, membrane protein, membrane protein, membrane protein, peptide ABC transporter/ATP-binding protein.	+	-	3/20	1/17	-
SP_1032-5	Iron-compound transporter system: iron-compound ABC transporter/iron compound-binding protein, iron-compound ABC transporter permease protein, iron-compound ABC transporter permease protein, iron-compound ABC transporter ATP-binding protein.	+	-	-	-	-
SP_1434-5	Transporter of drugs: transcriptional regulator AraC family, ABC transporter (permease/ATP-binding protein), ABC transporter (ATP-binding protein), hypothetical protein, conserved domain protein, ABC transporter (ATP-binding protein).	11/13	-	-	-	-
SP_1500	Glutamine ABC transporter substrate-binding protein	+	+	2/20 Two cluster III strains	5/17 All subsp. *dentisani*	-
SP_1704-6	ABC transporter/ATP-binding protein, membrane protein, membrane protein. Unknown function.	+	+	3/20	-	-
SP_1824-30	Phosphate ABC transporter: permease protein, ATP-binding protein, substrate-binding protein, hypothetical protein, UDP-glucose 4-epimerase, galactose-1-phosphate uridylyltransferase, phosphate transport system regulatory protein PhoU.	+	+	4/20	-	-
SP_2081-8	Phosphate transport system: conserved hypothetical protein, response regulator, histidine kinase PnpS, phosphate ABC transporter: phosphate-binding protein + 2 permease proteins, ATP-binding protein, phosphate transport system regulatory protein PhoU.	+	-	-	-	-
