# Supplementary material for: Identification of Virulence-Associated Properties by Comparative Genome Analysis of Streptococcus pneumoniae, S. pseudopneumoniae, S. mitis, Three S. oralis Subspecies, and S. infantis
Source: mBio. 2019 Sep 3;10(5):e01985-19. doi: 10.1128/mBio.01985-19 (PMC6722419; doi:10.1128/mBio.01985-19)

Figure S1. Summary of genome properties of the completed genomes of S. mitis NCTC12261 (type strain) and SK637. A. The outer rim displays a GC % bar graph in red (the dashed line shows the maximum value). The next two rims show CDSs predicted on the forward and reverse strands, green bars indicate rRNAs and red bars tRNAs. The next two rims (rims 4 and 5 from the outside) indicate core genes that are shared by all seven strains included in the display (including the reference) and unique genes that are present only in the reference genome (NCTC 12261 or SK637), respectively. The sixth innermost rims indicate genes that are shared by the strain noted on the rim and the reference strain. B. The two S. mitis genomes display a symmetrical GC-skew profile (G-C/G+C) typical of low-GC Gram-positive species where ~50% of the GC-skew function is positive starting at the dnaA gene and the other half is negative.

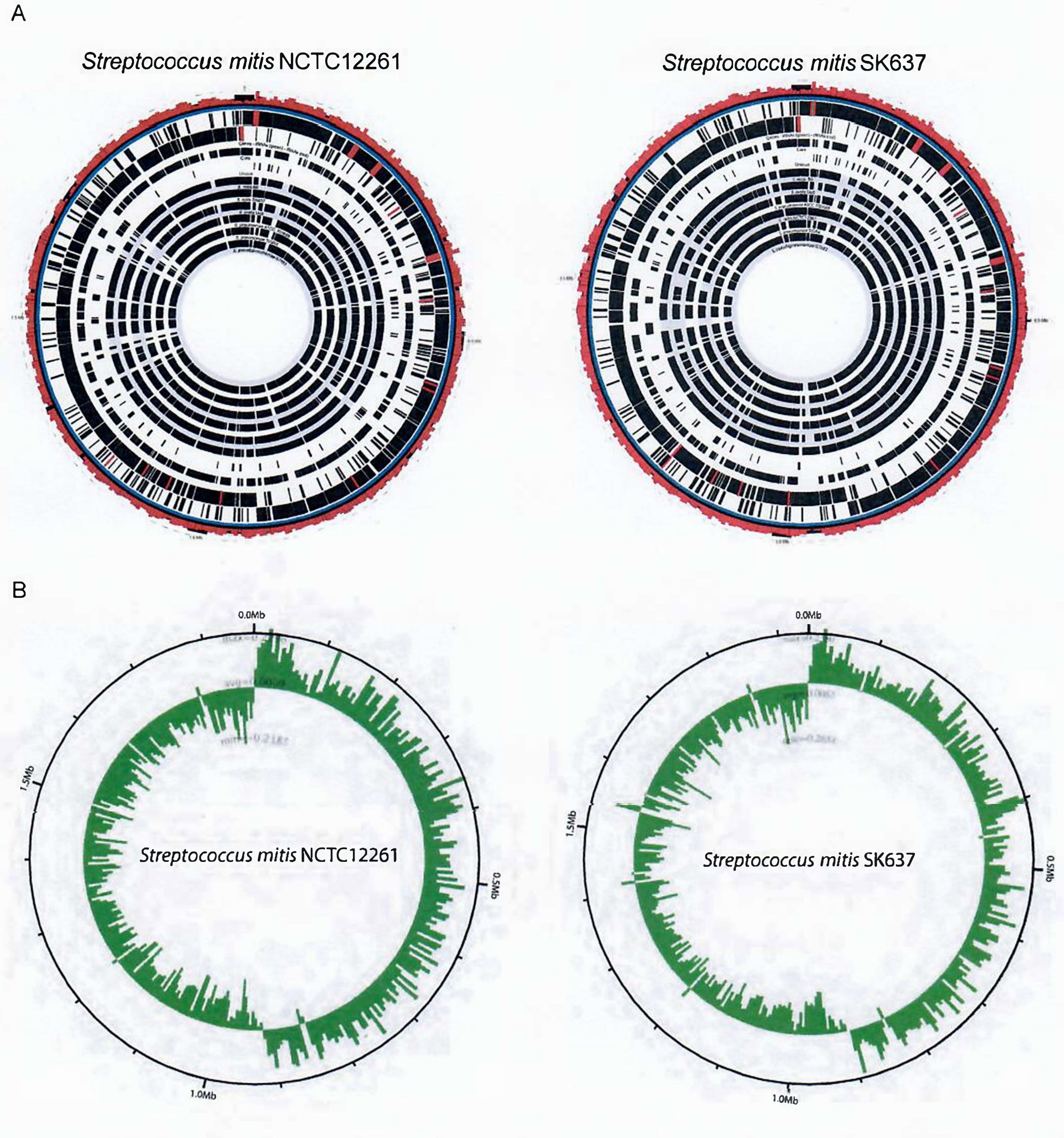

Supplement: FIG S1 [file mBio.01985-19-sf001.pdf]
