# Supplementary material for: Identification of Virulence-Associated Properties by Comparative Genome Analysis of Streptococcus pneumoniae, S. pseudopneumoniae, S. mitis, Three S. oralis Subspecies, and S. infantis
Source: mBio. 2019 Sep 3;10(5):e01985-19. doi: 10.1128/mBio.01985-19 (PMC6722419; doi:10.1128/mBio.01985-19)

Figure S2. Type 1 RM systems in streptococcal genomes. A. The type 1 RM system (tvr) spanning SP_0886 – SP_0892 in TIGR4 was present in all strains of *S. pneumoniae* and *S. pseudopneumoniae*, in some strains of the three *S. oralis* subspecies, and in *S. infantis* SK1302 (Table S2), but not in any of the *S. mitis* strains. The locus was interspersed with phage integrase genes, genes encoding an AbrB family transcriptional regulator and a death-on-curing protein and, in some strains, an additional restriction endonuclease or modification gene. B. The unrelated type I RM system in *S. mitis* NCTC12261 (SM12261_1408-1413) was present in the majority of commensal species except for strains of *S. pseudopneumoniae* and three strains of *S. mitis*. The locus showed a remarkable strain-specific organization and degree of completeness.

A

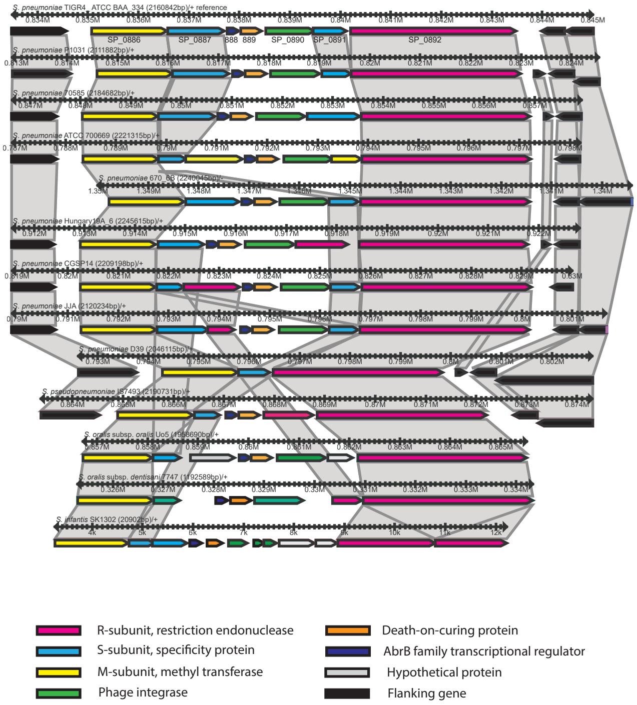

B

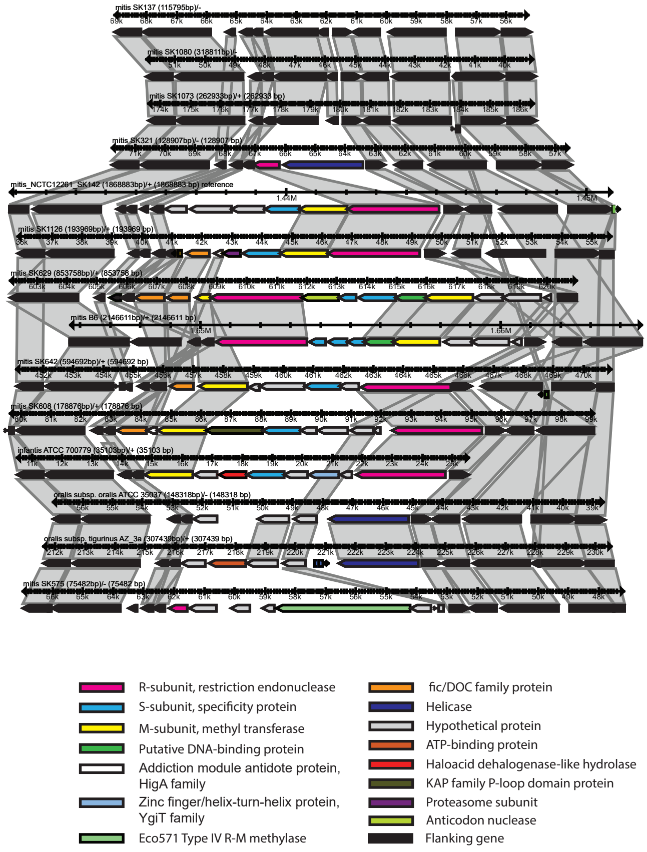

Supplement: FIG S2 [file mBio.01985-19-sf002.pdf]
