# Supplementary material for: Identification of Virulence-Associated Properties by Comparative Genome Analysis of Streptococcus pneumoniae, S. pseudopneumoniae, S. mitis, Three S. oralis Subspecies, and S. infantis
Source: mBio. 2019 Sep 3;10(5):e01985-19. doi: 10.1128/mBio.01985-19 (PMC6722419; doi:10.1128/mBio.01985-19)
Supplement: FIG S3 [file mBio.01985-19-sf003.pdf]

Table S3. Teichoic acid biosynthesis genes

S. pneumoniae TIGR4 locus	*S. neumoniae* R6 locus	*S. oralis* Uo5 locus	Gene symbol	Overall function	Enzyme function	*S. pneumoniae*		Presence %			
						TIGR4 C-polysacch/ O-antigen	serotype 5 70585	*S. pseudo-pneumoniae* N = 3	*S. mitis* N = 20	*S. oralis* N = 16	*S. infantis* N = 5
SP_0102	Spr0091	-		Synthesis and polymerization of repeating unit	Glycosyl transferase; addition of glucose to AATGal-*PP-upr*	+	-	100	15	0	0
SP70685_0164	-	Sor_1862		Synthesis and polymerization of repeating unit	Glycosyltransferase, family 2; presumably adds galactose (instead of glucose) to AAT-Gal-*PP-upr*	-	+	0	85	100	100
SP_0103	Spr0092	Sor_1861		Synthesis, activation and lipid-modification of AATGal	NAD dependent epimerase/dehydratase family protein; proposed role: synthesis of UDP-4-keto-6-deoxy-D-glucose from UDP-N-acetyl-glucosamine	+	+	100	100	100	100
SP_1267	Spr1145	Sor_1095	*licC*	Uptake and activation of choline	Cytidyltransferase; synthesis of CDP-choline	+	+	100	100	100	100
SP_1268	Spr1146	Sor_1096	*licB*		Choline transporter; import of choline	+	+	100	100	100	100
SP_1269	Spr1147	Sor_1097	*licA/pck*		Choline kinase; phosphorylation of choline	+	+	100	100	100	100
SP_1270	Spr1148	Sor_1098	*tarJ*	Synthesis and activation of ribitol	Cytidyltransferase; synthesis of cytidine 5'-di-phosphate (CDP)-ribitol from ribitol-5P and cytidine 5'-triphosphate	+	+	100	100	100	100
SP_1271	Spr1149	Sor_1099	*tarI*		NADPH-dependent ribulose-5P dehydrogenase; synthesis of ribitol-5P from ribulose-5P	+	+	100	100	100	100
SP_1272	Spr1150	Sor_0765	*tacF*	Flipping of subunits (or chains) across the membrane	Transmembrane transporter, flippase; proposed role: transport of the teichoic acid subunits across the cytoplasmic membrane	+	+	100	100	12.5	100
SP_1273	Spr1151	-	*licD1*	Loading of repeating units with PCho	Phosphotransferase; transfer of PCho to repeating unit	+	+	100	85	0	0
SP_1274	Spr1152	-	*licD2*		Phosphotransferase; transfer of PCho to repeating unit	+	+	100	85	0	0
SP_1364	Spr1222	-	*tarP*	Synthesis and polymerization of repeating unit	Integral membrane protein; proposed role: re-peating unit polymerase	+	+	100	80	0	0
SP_1365	Spr1223	-			Glycosyl transferase, group 2 family protein; proposed role: attachment of GalNAc or Glc	+	+	100	80	12.5	0
SP_1366	Spr1224	-			Glycosyl transferase, group 1 family protein; proposed role: attachment of GalNAc or Glc	+	+	100	80	12.5	20
SP_1367	Spr1225	Sor_0760	*licD3*		Phosphotransferase, LICD family protein; proposed role: transfer of ribitol-P to Glc in repeating unit synthesis	+	+	100	100	100	100
SP_1368	Spr1226	Sor_0759	*psr*	Transfer of subunits (or chains) from lipid-linked precursor to the cell wall	LCP family protein; Transfer of TA subunits to the peptidoglycan or to diacyl-glycerol	+	+	100	100	100	100
SP_1837	Spr1654	Sor_0469		Synthesis, activation and lipid-modification of AATGal	Aminotransferase class I and II; proposed role: synthesis of UDP-AATGal from UDP-4-keto-6-deoxy-D-glucose	+	+	100	100	100	100
SP_1838	Spr1655	Sor_0468			Bacterial sugar transferase; proposed role: transfer of AATGal from UDP-AATGal to upr-P	+	+	100	100	100	100
SP_1942	Spr1759	Sor_1707	*lytR*	Transfer of subunits (or chains) from lipid-linked precursor to the cell wall	pLCP family protein; Transfer of TA subunits to the peptidoglycan or to diacyl-glycerol	+	+	100	100	100	100
		Sor_0761		Synthesis and polymerization of repeating unit	Glycosyl-transferase, pfam00535 family; presumably transferring one of the GalNac residues to the repeating unit	-	-	0	0	37.5	0
		Sor_0762	*licD4*		Suggested to catalyze polymerization of TA precursors, and addition of phosphorylcholine	-	-	0	0	37.5	0
		Sor_0763			Suggested to add another phosphorylcholine residue to the repeating unit	-	-	0	0	37.5	0

S. pneumoniae TIGR4 locus	*S. oralis* Uo5 locus	*Streptococcus mitis*																			
		B6	NCTC 12261	SK137	SK271	SK321	SK564	SK569	SK575	SK578	SK579	SK597	SK608	SK616	SK629	SK637	SK642	SK667	SK1073	SK1080	SK1126
SP_0102	-	-	-	+	-	-	-	-	-	-	-	-	+	-	-	-	-	-	-	-	+
SP70685_0164	Sor_1862	+	+	-	+	+	+	+	+	+	+	+	-	+	+	+	+	+	+	+	-
SP_0103	Sor_1861	+	+	+	+	+	+	+	+	+	+	+	+	+	+	+	+	+	+	+	+
SP_1267	Sor_1095	+	+	+	+	+	+	+	+	+	+	+	+	+	+	+	+	+	+	+	+
SP_1268	Sor_1096	+	+	+	+	+	+	+	+	+	+	+	+	+	+	+	+	+	+	+	+
SP_1269	Sor_1097	+	+	+	+	+	+	+	+	+	+	+	+	+	+	+	+	+	+	+	+
SP_1270	Sor_1098	+	+	+	+	+	+	+	+	+	+	+	+	+	+	+	+	+	+	+	+
SP_1271	Sor_1099	+	+	+	+	+	+	+	+	+	+	+	+	+	+	+	+	+	+	+	+
SP_1272	Sor_0765	+	+	+	+	+	+	+	+	+	+	+	+	+	+	+	+	+	+	+	+
SP_1273	-	+	+	+	+	+	-	+	-	-	+	+	+	+	+	+	+	+	+	+	+
SP_1274	-	+	+	+	+	+	-	+	-	-	+	+	+	+	+	+	+	+	+	+	+
SP_1364	-	+	+	+	+	+	-	+	-	-	+	+	+	+	+	+	+	+	-	+	+
SP_1365	-	+	+	+	+	+	-	+	-	-	+	+	+	+	+	+	+	+	-	+	+
SP_1366	-	+	+	+	+	+	-	+	-	-	+	+	+	+	+	+	+	+	-		+
SP_1367	Sor_0760	+	+	+	+	+	+	+	+	+	+	+	+	+	+	+	+	+	+	+	+
SP_1368	Sor_0759	+	+	+	+	+	+	+	+	+	+	+	+	+	+	+	+	+	+	+	+
SP_1837	Sor_0469	+	+	+	+	+	+	+	+	+	+	+	+	+	+	+	+	+	+	+	+
SP_1838	Sor_0468	+	+	+	+	+	+	+	+	+	+	+	+	+	+	+	+	+	+	+	+
SP_1942	Sor_1707	+	+	+	+	+	+	+	+	+	+	+	+	+	+	+	+	+	+	+	+
	Sor_0761	-	-	-	-	-	-	-	-	-	-	-	-	-	-	-	-	-	-	-	-
	Sor_0762	-	-	-	-	-	-	-	-	-	-	-	-	-	-	-	-	-	-	-	-
	Sor_0763	-	-	-	-	-	-	-	-	-	-	-	-	-	-	-	-	-	-	-	-

S. pneumoniae TIGR4 locus	*S. oralis* Uo5 locus	*S. pseudopneumoniae*			*S. oralis* subsp. *oralis*						*S. oralis* subsp. *tigurinus*					*S. oralis* subsp. *dentisani*					*S. infantis*				
		ATCC BAA-960	IS7493	SK674	SK141	SK143	SK10	Uo5	SK23	SK610	AZ-3a	SK255	SK304	SK313	SK1074	7747	7746	F0392	F0407	SK95	ATCC 700779	SK1076	SK1302	SK970	SPAR10
SP_0102	-	+	+	-	-	-	-	-	-	-	-	-	-	-	-	-	-	-	-	-	-	-	-	-	-
SP70685_0164	Sor_1862	-	-	-	+	+	+	+	+	+	+	+	+	+	+	+	+	+	+	+	+	+	+	+	+
SP_0103	Sor_1861	+	+	+	+	+	+	+	+	+	+	+	+	+	+	+	+	+	+	+	+	+	+	+	+
SP_1267	Sor_1095	+	+	+	+	+	+	+	+	+	+	+	+	+	+	+	+	+	+	+	+	+	+	+	+
SP_1268	Sor_1096	+	+	+	+	+	+	+	+	+	+	+	+	+	+	+	+	+	+	+	+	+	+	+	+
SP_1269	Sor_1097	+	+	+	+	+	+	+	+	+	+	+	+	+	+	+	+	+	+	+	+	+	+	+	+
SP_1270	Sor_1098	+	+	+	+	+	+	+	+	+	+	+	+	+	+	+	+	+	+	+	+	+	+	+	+
SP_1271	Sor_1099	+	+	+	+	+	+	+	+	+	+	+	+	+	+	+	+	+	+	+	+	+	+	+	+
SP_1272	Sor_0765	+	+	+	+	+	-	+	-	+	+	+	+	+	+	+	+	+	+	+		+	+	+	+
SP_1273	-	+	+	+	-	-	-	-	-	-	-	-	-	-	-	-	-	-	-	-	-	-	-	-	-
SP_1274	-	+	+	+	-	-	-	-	-	-	-	-	-	-	-	-	-	-	-	-	-	-	-	-	-
SP_1364	-	+	+	+	-	-	-	-	-	-	?	?	-	-	-	-	-	-	-	-	-	?	-	-	-
SP_1365	-	+	+	+	-	-	-	-	-	-	+	+	-	-	-	-	-	-	-	-	-	-	-	-	-
SP_1366	-	+	+	+	-	-	-	-	-	-	+	+	-	-	-	-	-	-	-	-	-	+	-	-	-
SP_1367	Sor_0760	+	+	+	+	+	+	+	+	+	+	+	+	+	+	+	+	+	+	+	+	+	+	+	+
SP_1368	Sor_0759	+	+	+	+	+	+	+	+	+	+	+	+	+	+	+	+	+	+	+	+	+	+	+	+
SP_1837	Sor_0469	+	+	+	+	+	+	+	+	+	+	+	+	+	+	+	+	+	+	+	+	+	+	+	+
SP_1838	Sor_0468	+	+	+	+	+	+	+	+	+	+	+	+	+	+	+	+	+	+	+	+	+	+	+	+
SP_1942	Sor_1707	+	+	+	+	+	+	+	+	+	+	+	+	+	+	+	+	+	+	+	+	+	+	+	+
	Sor_0761	-	-	-	+	+	+	+	+	+	-	-	-	-	-	-	-	-	-	-	-	-	-	-	-
	Sor_0762	-	-	-	+	+	+	+	+	+	-	-	-	-	-	-	-	-	-	-	-	-	-	-	-
	Sor_0763	-	-	-	+	+	+	+	+	+	-	-	-	-	-	-	-	-	-	-	-	-	-	-	-
