# Supplementary material for: Identification of Virulence-Associated Properties by Comparative Genome Analysis of Streptococcus pneumoniae, S. pseudopneumoniae, S. mitis, Three S. oralis Subspecies, and S. infantis
Source: mBio. 2019 Sep 3;10(5):e01985-19. doi: 10.1128/mBio.01985-19 (PMC6722419; doi:10.1128/mBio.01985-19)

Figure S4. Phylogenetic analysis of neuraminidase/sialidase genes disclosing their presence in the individual taxa. In addition to nanA, B, and C, the analysis disclosed at least two additional clusters of putative sialidases with LPXTG cell-wall anchor motifs. These yet unnamed putative sialidases were present in all three strains of *S. pseudopneumoniae* and in selected strains of *S. mitis*, the individual strains having two distinct genes. The figures at individual nodes represent boot-strap values.

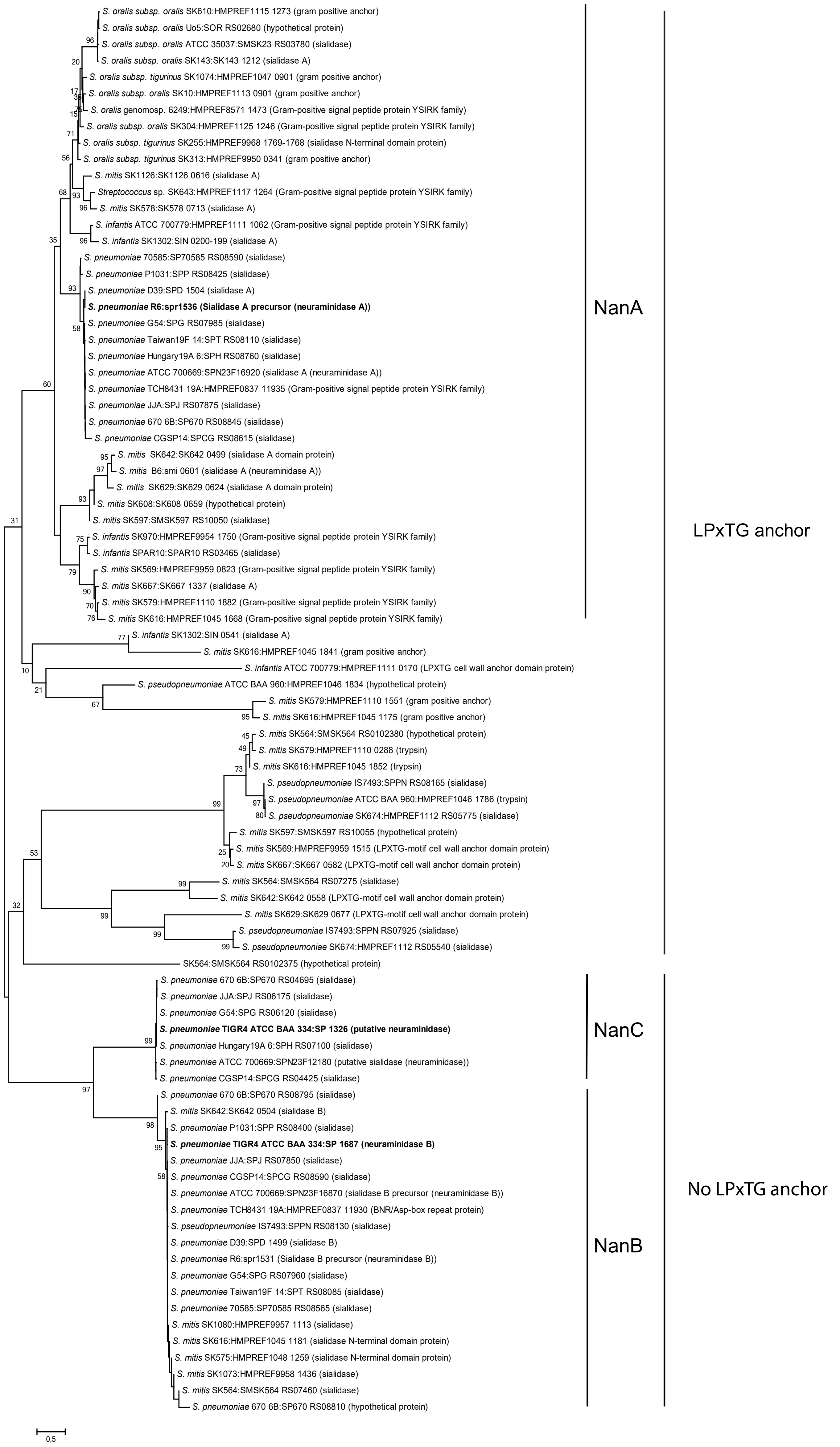

Supplement: FIG S4 [file mBio.01985-19-sf004.pdf]
