# Supplementary material for: Identification of Virulence-Associated Properties by Comparative Genome Analysis of Streptococcus pneumoniae, S. pseudopneumoniae, S. mitis, Three S. oralis Subspecies, and S. infantis
Source: mBio. 2019 Sep 3;10(5):e01985-19. doi: 10.1128/mBio.01985-19 (PMC6722419; doi:10.1128/mBio.01985-19)

Figure S5A. Mid-point rooted circular tree based on >7,500 streptococcal genome assemblies. *S. pneumoniae* strain labels are colored in black except for the 13 gap-free genomes that were selected for our detailed study that are shown in orange (n=12) or magenta (TIGR4) and labeled with large orange or magenta circles. *S. pseudopneumoniae* labels are blue, *S. mitis* red, *S. oralis* green, and *S. infantis* cyan. Entire non-pneumococcal clades were further highlighted using the same colors. Taxa that were erroneously assigned to a given species are easily identified by mismatched colors within each of the clades, with the exception of the *S. pneumoniae* clade that exclusively harbors *S. pneumoniae* strains.

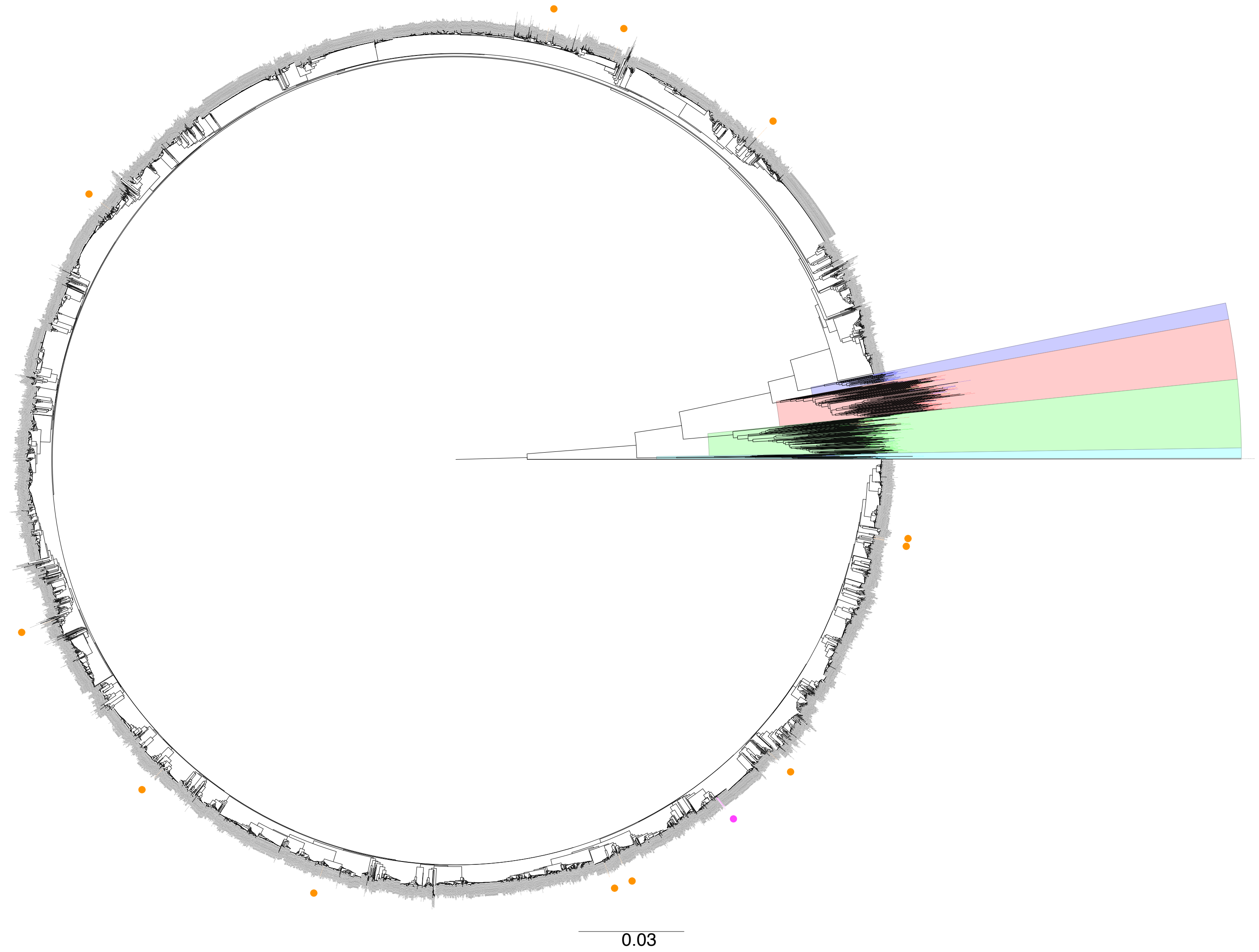

Figure S5B Splitstree phylogenetic analysis of 40 *S. mitis* genomes revealing multiple subclusters. Two genomes of *S. pneumoniae* were included in the analysis as references.

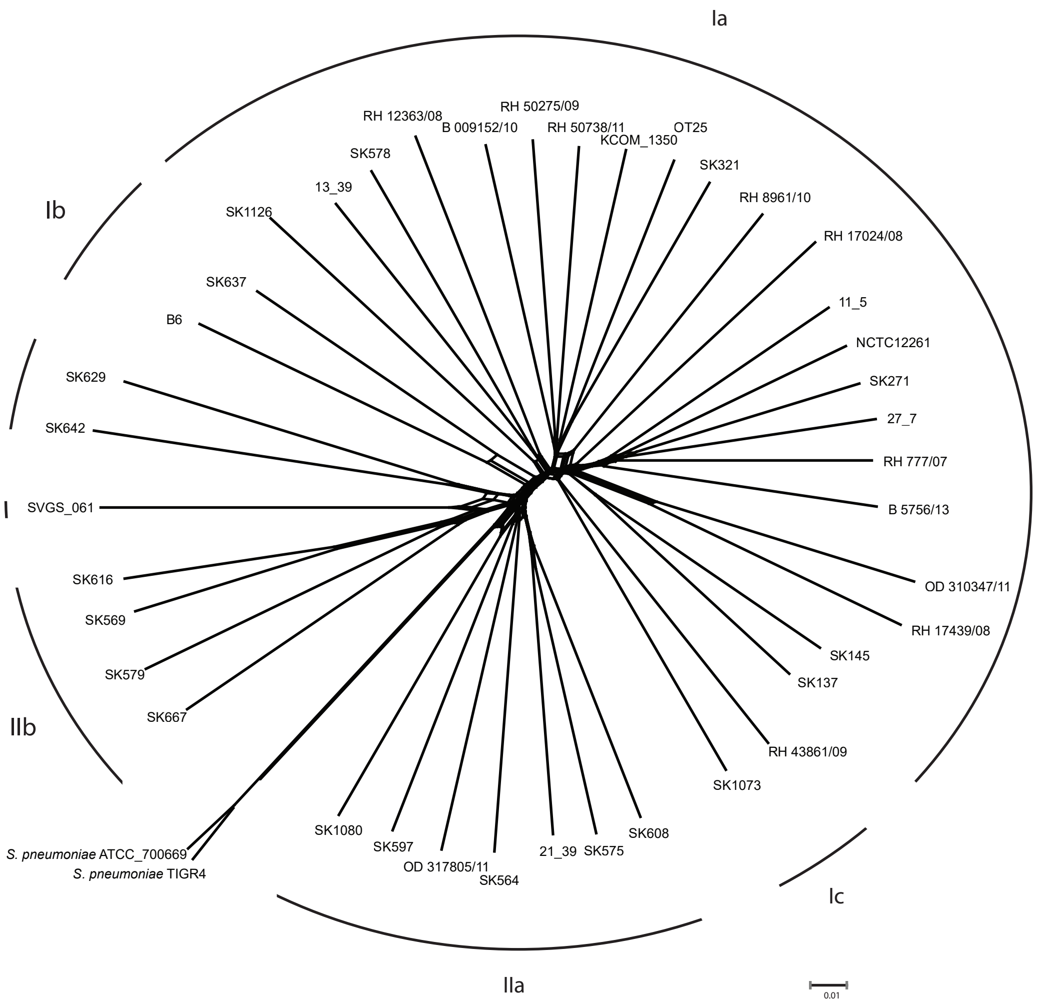

Supplement: FIG S5 [file mBio.01985-19-sf005.pdf]
